# An efficient approach for the green synthesis of biologically active 2,3-dihydroquinazolin-4(1*H*)-ones using a magnetic EDTA coated copper based nanocomposite†

**DOI:** 10.1039/d2ra07496f

**Published:** 2023-01-11

**Authors:** Sahil Kohli, Garima Rathee, Sunita Hooda, Ramesh Chandra

**Affiliations:** a Drug Discovery & Development Laboratory, Department of Chemistry, University of Delhi Delhi-110007 India rameshchandragroup@gmail.com acbrdu@hotmail.com; b Department of Chemistry, Acharya Narendra Dev College, University of Delhi Delhi-110019 India sunitahooda@andc.du.ac.in; c Dr B.R. Ambedkar Center for Biomedical Research (ACBR), University of Delhi Delhi-110007 India; d Institute of Nanomedical Science (INMS), University of Delhi Delhi-110007 India

## Abstract

2,3-Dihydroquinazolinone derivatives are known for antiviral, antimicrobial, analgesic, anti-inflammatory, and anticancer activities. However, recent approaches used for their synthesis suffer from various drawbacks. Therefore, we have fabricated a highly efficient magnetic EDTA-coated catalyst, Fe_3_O_4_@EDTA/CuI *via* a simple approach. The ethylenediamine tetraacetic acid (EDTA) plays a crucial role by strongly trapping the catalytic sites of CuI nanoparticles on the surface of the Fe_3_O_4_ core. The designed nanocatalyst demonstrates its potential for the catalytic synthesis of 2,3-dihydroquinazolinones using 2-aminobenzamide with aldehydes as the reaction partners. The nanocatalyst was thoroughly characterized through X-ray diffraction (XRD), Fourier-transform infrared spectroscopy (FTIR), vibrating sample magnetometry (VSM), transmission electron microscopy (TEM), scanning electron microscopy (SEM), energy dispersive X-ray analysis (EDX), X-ray photoelectron spectroscopy (XPS) and inductively coupled plasma analysis (ICP). The physiochemically characterized nanocatalyst was tested for synthesis of 2,3-dihydroquinazolinones and higher yields of derivatives were obtained with less time duration. Moreover, the catalytic synthesis is easy to operate without the use of any kind of additives/bases. Furthermore, the catalyst was magnetically recoverable after the completion of the reaction and displayed reusability for six successive rounds without any loss in its catalytic efficiency (confirmed by XRD, SEM, and TEM of the recycled material) along with very low leaching of copper (2.12 ppm) and iron (0.06 ppm) ions. Also, the green metrics were found in correlation with the ideal values (such as *E* factor (0.10), process mass intensity (1.10), carbon efficiency (96%) and reaction mass efficiency (90.62%)).

## Abstract 2

The fabricated catalyst Fe_3_O_4_@EDTA/CuI facilitates the synthesis of 2,3-dihydroquinazolin-4(1*H*)-ones under sustainable conditions with ideal values of green metrics in a short reaction time.

## Graphical abstract

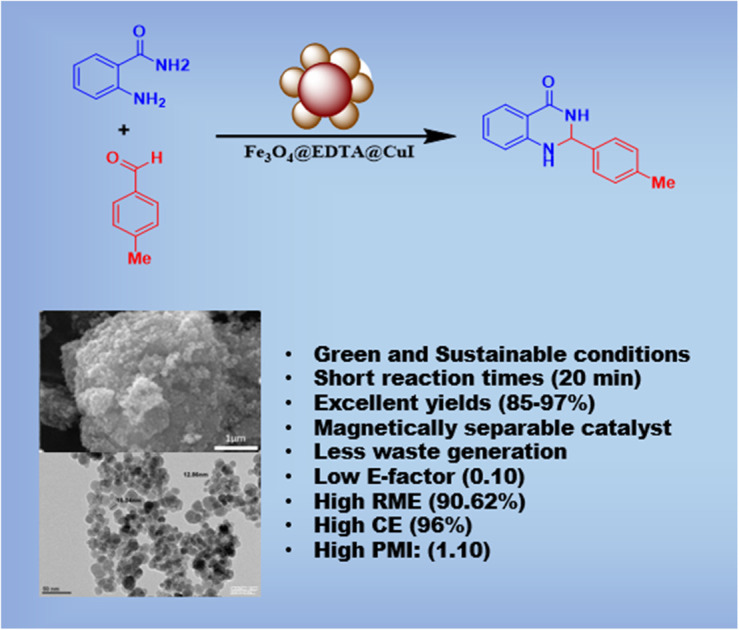

## 1. Introduction

Magnetic nanoparticles have gained great interest in the last few years due to their excellent properties such as low cost, low toxicity, high surface area to bulk ratios, high activity, thermal stability, and the surface modifications capability, easy dispersion, and superparamagnetic behavior.^1–3^ The modification of their surfaces with different amino ligands prevent them from aggregating which leads to stabilized active metal species over the surface. Magnetic recoverable catalysts have a wide range of uses in organic transformations such as C–H activation,^4^ coupling reactions,^5–7^ reduction reactions,^8^ oxidation reactions,^9^ and the synthesis of many heterocyclic compounds.^10,11^ Moreover, these magnetic nanoparticles can be separated from the reaction mixture very easily using a magnet. Magnetic separable catalysts come out to be a bridge between homogenous and heterogeneous catalysts.^12^ Copper is a 3d transition metal whose materials have been widely used in many catalytic reactions due to its various oxidation states from Cu^0^ to Cu^3+^. Also, copper-based nanomaterials have a high boiling point of 2562 °C, which marks them appropriate for high temperature and pressure conditions.^13–16^

The effective approach for the synthesis of highly robust, selective and low-cost copper based nanoparticles is to create magnetic copper based nanocomposites which have been used in several organic transformation due to their easy separation with low leaching of copper.^17–21^ Fe_3_O_4_ and copper composites have found many application in many organic transformation such as synthesis of propargylamines using Cu(i)-pybox–Fe_3_O_4_ nanocomposites, Fe_3_O_4_@PmPDs@Cu_2_O as a nanocatalyst in the synthesis of 5-phenyl-[1,2,3]triazolo[1,5-*c*]quinazolines, Cu@DOPA@Fe_3_O_4_ as a nanocatalyst in the cross coupling of thiols and aryl halides, Fe_3_O_4_@CS-TCT-Tet-Cu(ii) nanocatalyst in the synthesis of *N*-sulfonyl-*N*-aryl tetrazoles and 5-arylamino-1*H*-tetrazoles *etc.*^22^ The development of many heterogeneous catalytic system involving metal–organic frameworks and nanoparticles for the synthesis of various nitrogen containing heterocycles have been extensively reported.^23^

**Fig. 1:**
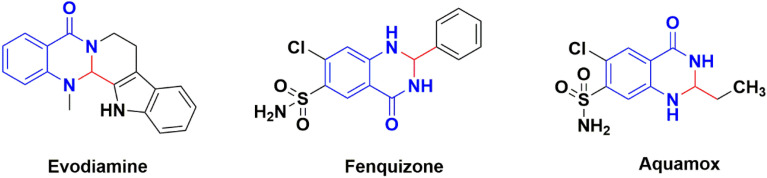
Biologically active molecules with 2,3-dihydroquinazolin-4(1*H*)-ones scaffolds.

2,3-Dihydroquinazolinones are essential nitrogen-containing heterocyclic moieties showing various biological and pharmacological activities such as antiviral, analgesic, anti-inflammatory, anticancer and antimicrobial (Fig. 1).^24–28^ Numerous procedures are known for synthesizing 2,3-dihydroquinazolin-4(1*H*)-ones like reductive cyclization of 2-azidobenzamides or 2-nitrobenzamides,^29^ quinazolin-4(3*H*)-ones reduction,^30^ condensations of 2-aminobenzamides with benzil,^31^ desulfurizations of 2-thioxoquinazolin-4(3*H*)-ones.^32^ While, the most common method involves the condensation of ketones or aldehydes with 2-aminobenzamide in the presence of various acid catalysts like cellulose-SO_3_H,^33^ cerium(iv) ammonium nitrate (CAN),^34^ p-TSA,^35^ Y(NO_3_)_3_,^36^ succinimide-*N*-sulfonic acid,^37^ and ZrCl_4_.^38^ However, these methods have various limitations which includes yields in low amounts, long reaction time, use of harmful solvents, complex work-up procedure, using expensive reagents and lack of catalyst reusability. Therefore, it is essential to develop a simple, efficient, green, and sustainable route to synthesize 2,3-dihydroquinazolinones.

Our research group has earlier successfully fabricated multiple nanocatalysts to synthesize numerous biologically active organic frameworks such as benzimidazoles, polyhydroquinolines, xanthenes, 1,4-dihydropyridines, and 4*H*-pyrans.^39–41^ In the present work, we design a novel, efficient, and magnetically recoverable Fe_3_O_4_@EDTA/CuI nanocatalyst to synthesize 2,3-dihydroquinazolin-4(1*H*)-ones through condensation of 2-aminobenzamide with different aldehydes. The surface of Fe_3_O_4_ has been modified with ethylenediaminetetraacetic acid (EDTA) *via* the wet chemical method, and then the CuI is supported over the modified surface of the magnetic nanoparticles. The techniques like FTIR, XRD, EDX, SEM, XPS, VSM, ICP and TEM were used to characterize the nanocatalyst. The fabricated material was relatively stable under the performed reaction conditions giving an excellent yield of the products, and was separated quite easily and reused for six times without much decrease in the % yield.

## 2. Results and discussion

### 2.1 Design and synthesis of the nanocatalyst

**Fig. 2:**
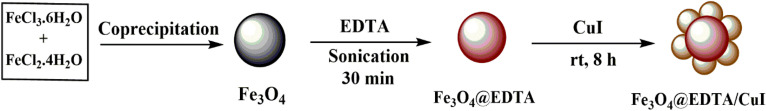
Schematic illustration of the steps involved in the fabrication of Fe_3_O_4_@EDTA/CuI.

The design and synthesis of the catalyst are shown in Fig. 2. It involves the synthesis of Fe_3_O_4_ nanoparticles by co-precipitation method followed by modification of the surface with EDTA, and further CuI was immobilized on the modified support resulting in Fe_3_O_4_@EDTA/CuI.

**Fig. 3:**
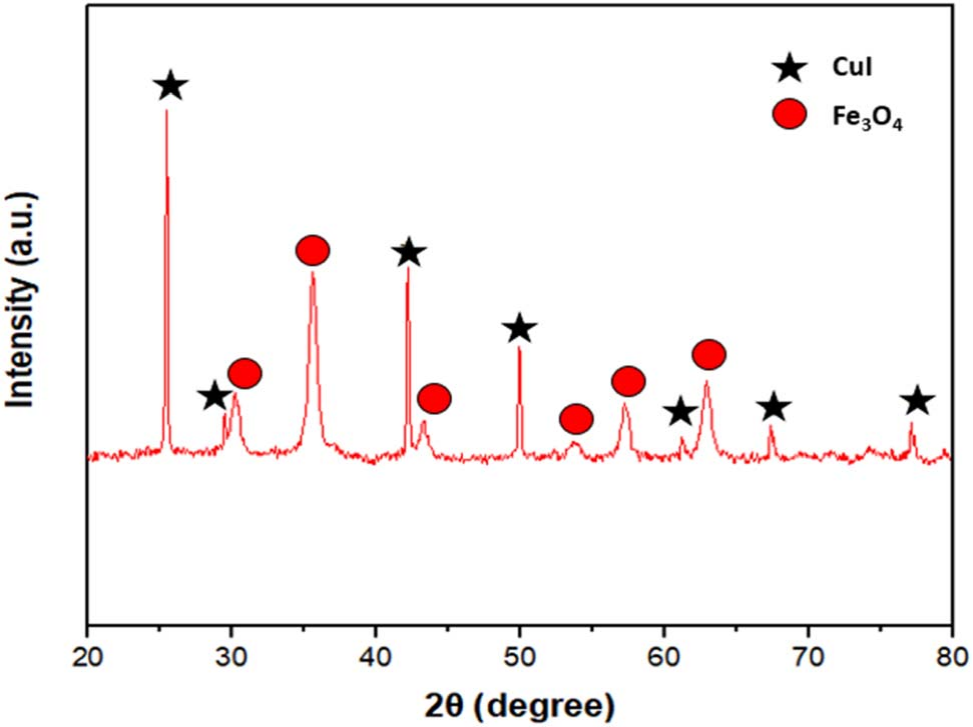
XRD pattern of nanocatalyst.

Fig. 3 shows the XRD spectra of Fe_3_O_4_@EDTA/CuI nanocatalyst. The peaks appearing at a value of 2*θ* = 30.2, 35.8, 43.4, 53.7, 57.8 and 63.1 corresponding to (220), (311), (400), (422), (511), and (440) is in good agreement with standard patterns of Fe_3_O_4_ whereas the peaks at 2*θ* = 25.5, 29.4, 42.3, 50.0, 61.3, 67.4 and 77.2 corresponds to (111), (200), (220), (311), (400), (331) and (422) of copper iodide which is also in agreement with the JCPDF file (06-0246).

**Fig. 4:**
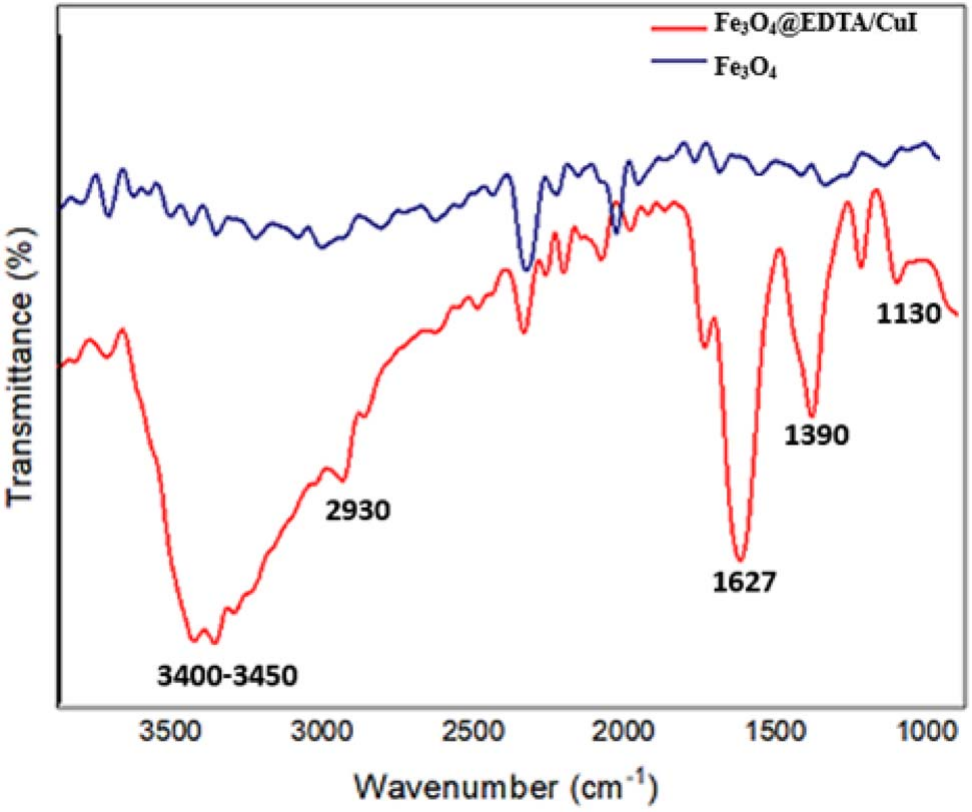
FTIR Spectra of the nanocatalyst.

Fig. 4 displays the FT-IR spectra of the magnetic Fe_3_O_4_@EDTA/CuI nanoparticles in the range of 400–4000 cm^−1^. The peaks observed at 1090, 1390, 1627, 2930 and 3400–3450 cm^−1^ related to the aliphatic C–N stretching, strong C–C–H stretching, C=O stretch vibration, C–H stretching, O–H vibration stretching, respectively, confirms the existence of EDTA on the surface of Fe_3_O_4_.^42,43^

**Fig. 5:**
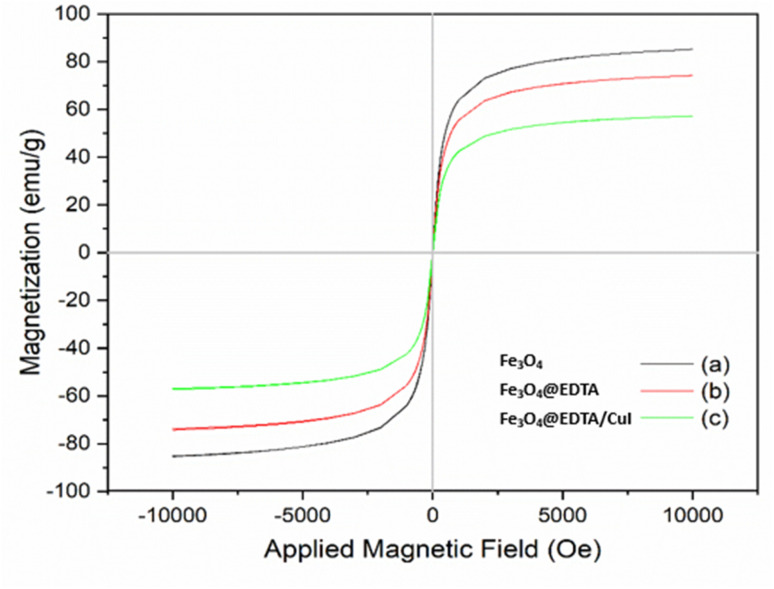
VSM curves of Fe_3_O_4_ (a), Fe_3_O_4_@EDTA (b) and Fe_3_O_4_@EDTA/CuI (c) nanoparticles.

Fig. 5 shows the magnetic behavior of Fe_3_O_4_ (a), Fe_3_O_4_@EDTA (b), and Fe_3_O_4_@EDTA–CuI evaluated by VSM at room temperature. The saturation magnetizations (*M*_s_) of the magnetic Fe_3_O_4_, Fe_3_O_4_@EDTA, and Fe_3_O_4_@EDTA/CuI were found to be 85.20, 74.25 and 57.20 emu g^−1^ respectively. There is a decrease in *M*_s_ value when EDTA is coated over Fe_3_O_4,_ and the value further decreases when CuI is immobilized over Fe_3_O_4_@EDTA. Also, the external magnet can easily separate the catalyst from the reaction mixture. Moreover, there were no coercivity, hysteresis loop, and remanence detected in any of the prepared nanomaterials, which shows the superparamagnetic nature of all.

**Fig. 6:**
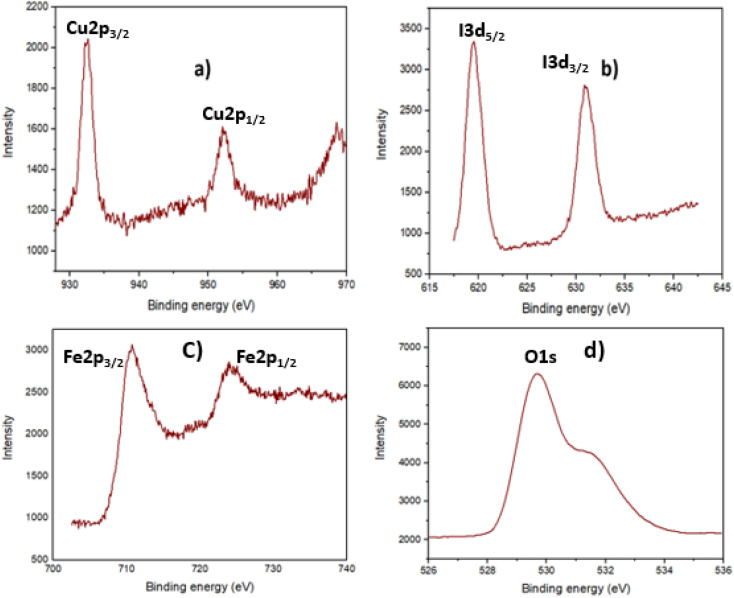
XPS spectrum of Fe_3_O_4_@EDTA/CuI in the region (a) Cu 2p (b) I 3d (c) Fe 2p (d) O 1 s.

Furthermore, CuI existence in the Fe_3_O_4_@EDTA/CuI nanocatalyst was also confirmed by X-ray photoelectron spectroscopy as shown in Fig. 6. The peak binding values at 932.3 eV and 952.2 eV were related to Cu 2p and the values at 619 eV and 632 eV were related to I 3p. The value resembles the reported data of CuI nanoparticles which also confirms the +1 oxidation state of copper.^44^ The values at 710.8 and 724.3 eV are allocated to the spin–orbit split doublet of Fe 2p_1/2_ and Fe 2p_3/2_ respectively which resembles with the reported values of Fe_3_O_4_.^45^ The broadness of the Fe 2p peaks confirms the presence of both oxidation states of iron (Fe^2+^ and Fe^3+^).^46^ The peak at 529.8 eV corresponds to O 1s peak of Fe_3_O_4_.

**Fig. 7:**
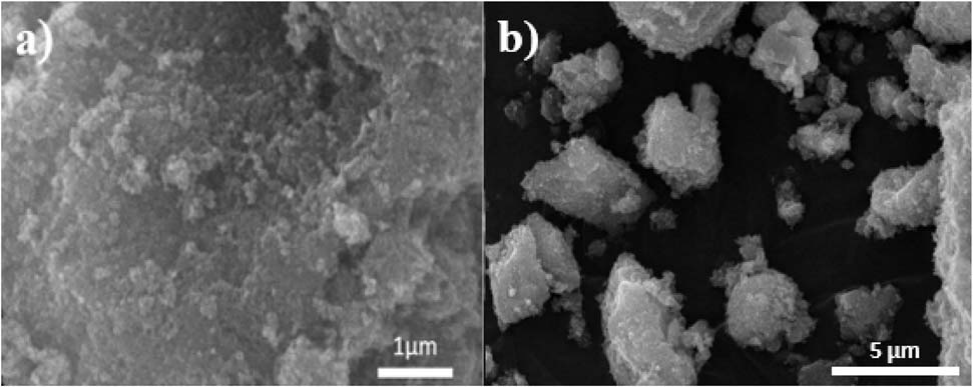
SEM of Fe_3_O_4_@EDTA/CuI.

**Fig. 8:**
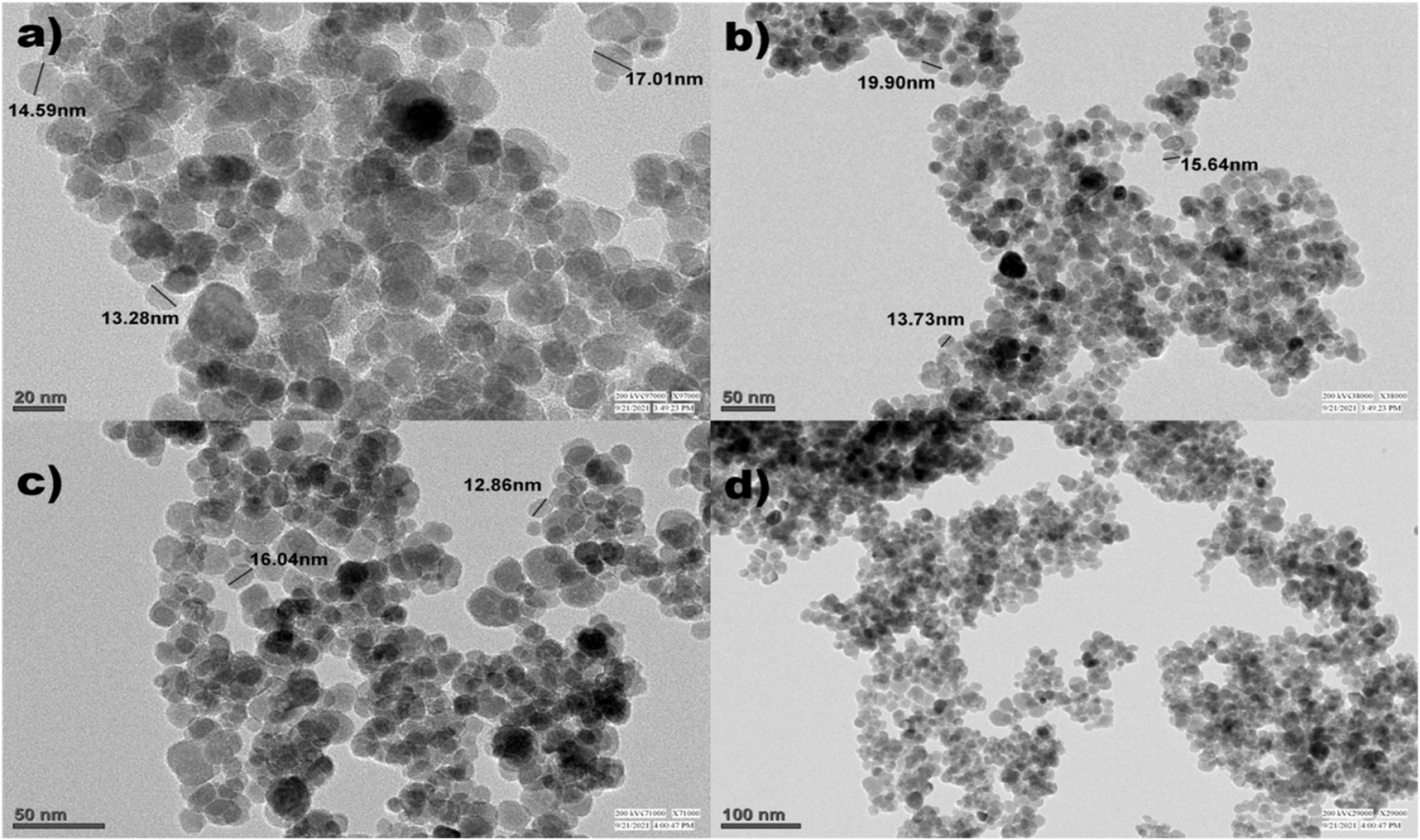
TEM of Fe_3_O_4_@EDTA/CuI.

Fig. 7 and 8 show the SEM and TEM images of Fe_3_O_4_@EDTA/CuI which clearly shows the accumulation of CuI over EDTA modified spherical Fe_3_O_4_ nanoparticles. The SEM images show Fe_3_O_4_@EDTA/CuI nanoparticles agglomerate into larger aggregates. The 20 nm, 50 nm, and 100 nm TEM images determine the synthesized material morphology and size. The images show spherical nanoparticles formation with 12–20 nm of the mean size range.

**Fig. 9:**
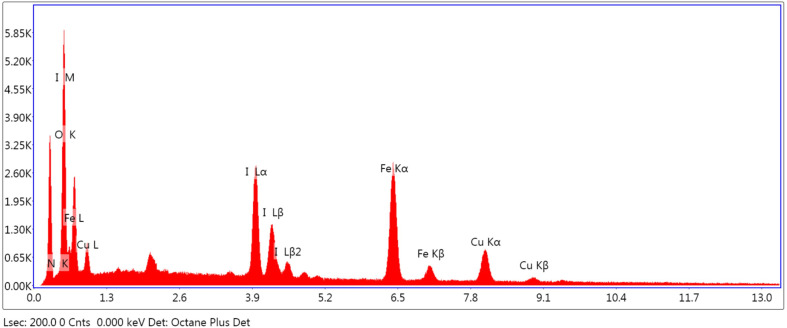
EDX of Fe_3_O_4_@EDTA/CuI nanocatalyst.

Fig. 9 displays the catalyst EDX spectra that clearly show the presence of Cu and I over Fe_3_O_4_@EDTA surface and the percentage composition of copper, iodine, iron, oxygen and nitrogen are 12.61 19.41, 22.44, 26.04, and 19.5 wt%, respectively.

### 2.2 Synthesis of 2,3-dihydroquinazolin-4(1*H*)-ones *via* Fe_3_O_4_@EDTA/CuI catalyst

Primarily, the *o*-aminobenzamide (1), *p*-methylbenzaldeyde (2a) and Fe_3_O_4_@EDTA/CuI catalyst (20 mg) were used in different solvents and neat condition (Table 1). It was observed that no product was obtained when toluene was used (entry: 1, Table 1). Then solvents such as DMF, DMSO, THF and acetonitrile, which are polar aprotic, were used. It was found that the product formed was 43%, 56%, 11%, and 23% in DMF, DMSO, acetonitrile, and THF respectively (entries: 2–5, Table 1).

**Table 1:** Optimization study for the nanocatalytic synthesis of 2,3-dihydroquinazolin-4(1*H*)-ones by using *o*-aminobenzamide (1) and *p*-methylbenzaldehyde (2a) as precursors and Fe_3_O_4_@EDTA/CuI as nanocatalyst^a^

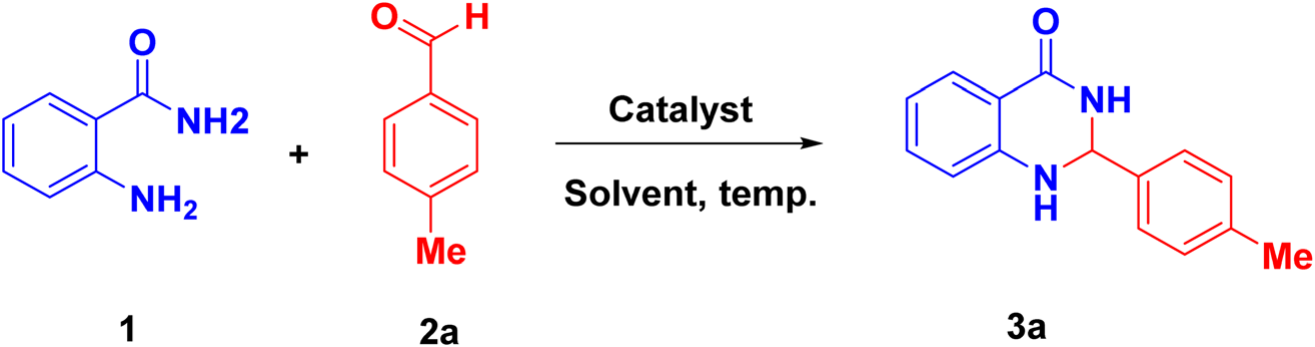					
Entry	Catalyst (mg)	Solvent	Temp (°C)	Time (min)	Yield (%)
1	Fe_3_O_4_@EDTA/CuI (20)	Toluene	60	60	—
2	Fe_3_O_4_@EDTA/CuI (20)	DMF	60	60	43
3	Fe_3_O_4_@EDTA/CuI (20)	DMSO	60	60	56
4	Fe_3_O_4_@EDTA/CuI (20)	Acetonitrile	60	60	23
5	Fe_3_O_4_@EDTA/CuI (20)	THF	60	60	11
6	Fe_3_O_4_@EDTA/CuI (20)	Water	60	60	—
7	Fe_3_O_4_@EDTA/CuI (20)	Neat	60	60	—
8	Fe_3_O_4_@EDTA/CuI (20)	EG	60	60	58
9	Fe_3_O_4_@EDTA/CuI (20)	Ethanol	60	20 min	62
10	Fe_3_O_4_@EDTA/CuI (20)	Methanol	Reflux	20 min	78
11	**Fe**_**3**_ **O**_**4**_ **@EDTA/CuI (20)**	**Ethanol**	**Reflux**	**20 min**	**97**
12	Fe_3_O_4_@EDTA/CuI (20)	Ethanol	Reflux	10 min	76
13	Fe_3_O_4_@EDTA/CuI (20)	Ethanol	Reflux	30 min	97
14	Fe_3_O_4_@EDTA/CuI (10)	Ethanol	Reflux	20 min	81
15	Fe_3_O_4_@EDTA/CuI (30)	Ethanol	Reflux	20 min	97
16	CuI (20)	Ethanol	Reflux	20 min	56

a Reaction condition: *o*-benzamide 1 (0.5 mmol), aldehyde 2 (0.5 mmol), Fe_3_O_4_@EDTA/CuI (10–30 mg) and solvent (3 mL) were stirred at appropriate temperature.

When solvents like EG (ethylene glycol), water, methanol, ethanol were used, no reaction in water was observed (entry: 6, Table 1); while using ethanol and EG, the product formed was 62% and 58% respectively (entry: 8–9, Table 1). Also, no product was formed in neat reaction conditions (entry: 7, Table 1). Then the reaction was refluxed in methanol and ethanol. The yields were 78% and 97% respectively (entry: 10–11, Table 1). The better appropriate solvent which affords the product was ethanol (entry: 11, Table 1). The effect of reaction time on yield was checked, and it was found to be 76% and 97% after 10 min and 30 min (entry: 12–13, Table 1). The effect of catalyst was also investigated, which shows that on decreasing and increasing the amount of catalyst, the yield observed was 81 and 97% (entry: 14–15, Table 1). Hence, no change was observed in yield on increasing the catalyst amount. When CuI was used, the yield obtained was 56% (entry: 16, Table 1). Hence, the appropriate reaction condition for nanocatalytic synthesis was 20 mg of Fe_3_O_4_@EDTA/CuI refluxed for 20 min in ethanol.

**Scheme 1:**
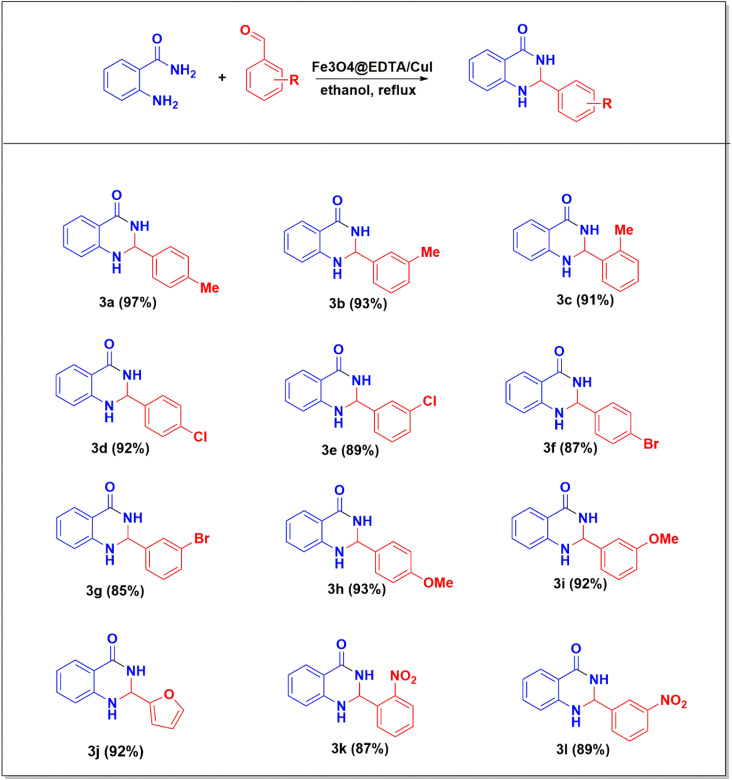
Fe_3_O_4_@EDTA/CuI catalyzed synthesis of 2,3-dihydroquinazolin-4(1*H*)-ones derivatives.^a a^Reaction condition: *o*-benzamide 1 (0.5 mmol), substituted aldehyde 2 (0.5 mmol), Fe_3_O_4_@EDTA/CuI (20 mg) and ethanol (3 mL) were refluxed for 20 min.

Derivatives of 2,3-dihydroquinazolin-4(1*H*)-ones were synthesized using the most appropriate reaction conditions as shown in Scheme 1. The excellent yield of the products were obtained in all from the various benzaldehydes (3a–3l).

**Fig. 10:**
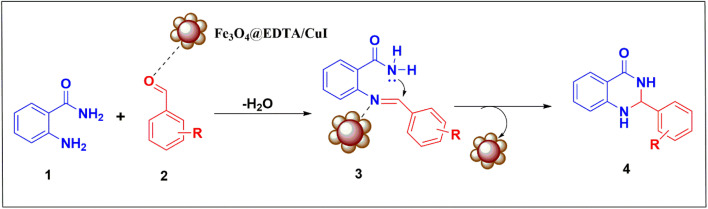
Plausible mechanism.

Fig. 10 shows the plausible mechanism for synthesizing 2,3-dihydroquinazolin-4(1*H*)-ones by Fe_3_O_4_@EDTA/CuI nanocatalyst. The first step involves reaction between 2-aminobenzamide (1) and aldehyde (2) in the presence of catalyst, where catalyst behaves as Lewis acid and interacts with oxygen atom of the carbonyl to increase the electrophilicity of aldehydic carbon to give Schiff base (3) by elimination of water molecule. The next step involves the amide nitrogen attack on electrophilic carbon of the imine followed by proton transfer to obtain 2,3-dihydroquinazolin-4(1*H*)-ones (4).

**Fig. 11:**
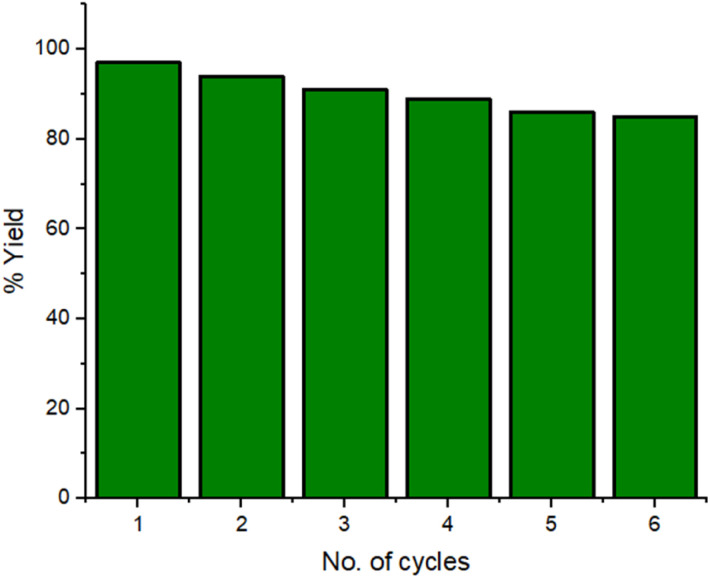
Recyclability of Fe_3_O_4_@EDTA/CuI nanocatalyst.

Then, we investigated the recyclability of the catalyst. The reaction was setup on a large scale using 5 mmol of reactants and 200 mg of catalyst. At the completion of every reaction cycle, the external magnet helped in separating the catalyst from the reaction mixture. The catalyst was washed with water and ethanol, dried and reused for subsequent reactions. The catalyst was reused six more successions and yield of the product was found to be 83% after sixth catalytic cycle as shown in Fig. 11. The stability of the material was checked after six runs by XRD, SEM and TEM which indicates that the structure and morphology of the catalyst remain unchanged (ESI Fig. S1–S3†). The ICP analysis of the filtrate was carried out after the removal of the catalyst from the reaction mixture and it was found that leached metal ion concentrations for copper and iron ion are 2.12 ppm and 0.06 ppm respectively which are lower than the authentic concentration of respective ions as per WHO terms.^47^

The calculative values of green metrics are shown in Table 2 (detail calculations in ESI†). The current method is green and sustainable as the green metrics are near ideal values. Also, the present catalyst shows better value of metrics than the previous reported methods.

**Table 2:** Comparison of green metrics with previously reported catalyst

S. No	Catalyst	*E* factor	Process mass intensity	Carbon efficiency	Reaction mass efficiency
1	Fe_3_O_4_@EDTA/CuI	0.10	1.10	96%	90.62%
2 (ref. 36)	Y(NO_3_)_3_	16.56	17.56	93%	75%
3 (ref. 47)	Gr@SO_3_H	0.16	1.16	92%	85.54%
4 (ref. 49)	g-C_3_N_4_@SO_3_Ch	0.31	1.31	82%	76.17%

Table 3 shows the comparison of Fe_3_O_4_@EDTA/CuI catalyst with various catalysts for the synthesis of 2-(4-methylphenyl)-2,3-dihydroquinazolin-4(1*H*)-one. It can be seen that the current catalyst has better reaction conditions when compared with other various catalysts.

**Table 3:** Comparison of the catalytic efficiency of the Fe_3_O_4_@EDTA/CuI with other various reported catalysts for the synthesis 2-(4-methylphenyl)-2,3-dihydroquinazolin-4(1*H*)-one

Entry	Catalyst	Solvent/condition	Time (min)	Yield (%)	Ref.
1	Cu(i)-modified-SBA-15	CH_2_Cl_2_, rt	90	94%	48
2	Gr@SO_3_H	Ethanol, reflux	40	92%	49
3	[Ce(L-Pro)_2_]_2_ (oxa)	Ethanol, 50–55 °C	240	87%	50
4	g-C_3_N_4_@SO_3_Ch	PEG, rt	140	82%	51
5	Y(NO_3_)_3_·6H_2_O	CH_3_CN, rt	300	96%	35
6	Fe_3_O_4_@EDTA/CuI	Ethanol, reflux	20	98%	This study

## 3. Experimental section

### 3.1 Preparation of Fe_3_O_4_, Fe_3_O_4_@EDTA, and Fe_3_O_4_@EDTA/CuI nanocomposites

The Fe_3_O_4_ nanoparticles were prepared by co-precipitation method. Briefly, 5.6 g of FeCl_3_·6H_2_O and 2.3 g of FeCl_2_·4H_2_O were dispersed in 100 mL of distilled water and stirred for 1 hour at 60 °C. Furthermore, 10 mL of ammonia (25%) solution was added dropwise with continuous stirring. The color changed instantly to black, and further, the reaction was stirred for 1 hour at 60 °C. The Fe_3_O_4_ nanoparticles were separated magnetically and washed with water and ethanol four times to remove any impurities. Lastly, the material was dried in the oven at 60 °C for 12 hours.

In 100 mL of distilled water, 1 g of synthesized Fe_3_O_4_ was dispersed with 1 g of 2Na-EDTA, and the mixture was sonicated for 30 min. The obtained Fe_3_O_4_@EDTA was isolated magnetically, washed with water followed by ethanol, and then dried in a vacuum oven at 60 °C for 12 hours.

500 mg of Fe_3_O_4_@EDTA was dispersed in 20 mL of distilled water and then 100 mg of CuI was added, and the mixture was stirred for 2 hours at room temperature. The final product was collected using a magnet and washed with distilled water and ethanol many times to eliminate impurities. The obtained catalyst Fe_3_O_4_@EDTA/CuI was dried at 60 °C for 12 hours.

### 3.2 General procedure for the synthesis of 2,3-dihydroquinazolin-4(1*H*)-ones

For the reaction, 0.5 mmol of aldehyde, 0.5 mmol of 2-aminobenzamide, 3 mL of ethanol and 20 mg of catalyst were added in a round bottom flask, and the mixture was refluxed under continuous stirring for the given time. The reaction was constantly monitored with the help of thin-layer chromatography. On completion of the reaction, the magnet was used to separate the catalyst from the reaction mixture. The product was purified using column chromatography to afford the final pure product.

## 4. Conclusion

In this work, we have developed a novel and efficient Fe_3_O_4_@EDTA/CuI catalyst to synthesize biologically interesting molecule 2,3-dihydroquinazolin-4(1*H*)-ones by reaction between 2-aminobenzamide and different aldehydes under green conditions. The method includes advantages like short reaction time, high yield, ambient reaction conditions, no additives, greener pathway, and the excellent value of green chemistry metrics. Furthermore, a magnet can easily collect the nanocatalyst and reuse it six more times with a very slight reduction in its catalytic action.

## 5. Spectral data

### 5.1 2-(4-Methylphenyl)-2,3-dihydroquinazolin-4(1*H*)-one (3a)

White solid; yield: 97%; mp: 230–232 °C; ^1^H NMR (DMSO-*d*_6_, 400 MHz): *δ* 8.21 (s, 1H), 7.58–7.56 (d, *J* = 9.21 Hz, 1H), 7.35–7.33 (d, *J* = 8.11 Hz, 2H), 7.22–7.14 (m, 3H), 7.03 (s, 1H), 6.72–6.61 (m, 2H), 5.67 (s, 1H), 2.25 (s, 3H). ^13^C NMR (DMSO-*d*_6_, 100 MHz): *δ* 164.19, 148.45, 139.17, 138.25, 133.79, 129.34, 127.87, 127.33, 117.60, 115.52, 114.94, 66.92, 21.26. Anal. calcd for C_15_H_14_N_2_O: C, 75.61; H, 5.92; N, 11.76; found C, 75.58; H, 5.94; N, 11.77.

### 5.2 2-(3-Methylphenyl)-2,3-dihydroquinazolin-4(1*H*)-one (3b)

Liquid; yield: 93%; ^1^H NMR (DMSO-*d*_6_, 400 MHz): *δ* 8.19 (s, 1H), 7.59–7.57 (d, *J* = 7.70 Hz, 1H), 7.28–7.12 (m, 5H), 7.03 (s, 1H), 7.35–7.33 (d, *J* = 8.11 Hz, 2H), 6.72–6.70 (d, *J* = 7.97 Hz, 1H), 6.65–6.62 (t, *J* = 7.83 Hz, 1H), 5.68 (s, 1H). ^13^C NMR (DMSO-d_6_, 100 MHz): *δ* 164.15, 148.44, 142.04, 137.95, 133.82, 129.61, 128.76, 128.03, 124.55, 117.61, 115.45, 114.91, 67.17, 21.60. Anal. calcd for C_15_H_14_N_2_O: C, 75.61; H, 5.92; N, 11.76; found C, 75.64; H, 5.93; N, 11.79.

### 5.3 2-(2-Methylphenyl)-2,3-dihydroquinazolin-4(1*H*)-one (3c)

Liquid; yield: 91%; ^1^H NMR (DMSO-*d*_6_, 400 MHz): *δ* 8.08 (s, 1H), 7.69–7.67 (d, *J* = 7.56 Hz, 1H), 7.59–7.58 (d, *J* = 6.60 Hz, 1H), 7.27–7.21 (m, 4H), 6.88 (s, 1H), 6.79–6.69 (s, 2H), 6.02 (s, 1H). ^13^C NMR (DMSO-*d*_6_, 100 MHz): *δ* 164.70, 149.12, 138.62, 136.68, 133.81, 131.25, 129.05, 128.02, 126.49, 117.81, 115.46, 115.07, 65.29, 19.35. Anal. calcd for C_15_H_14_N_2_O: C, 75.61; H, 5.92; N, 11.76; found C, 75.62; H, 5.89; N, 11.72.

### 5.4 2-(4-Chlorophenyl)-2,3-dihydroquinazolin-4(1*H*)-one (3d)

White solid; yield: 92%; mp: 197–199 °C; ^1^H NMR (DMSO-*d*_6_, 400 MHz): *δ* 8.33 (s, 1H), 7.59–7.57 (d, *J* = 7.56 Hz, 1H), 7.49–7.41 (m, 4H), 7.24–7.19 (m, 1H), 7.12 (s, 1H), 6.73–6.71 (d, *J* = 8.11 Hz, 1H), 6.66–6.63 (t, *J* = 7.56 Hz, 1H), 5.75 (s, 1H). ^13^C NMR (DMSO-*d*_6_, 100 MHz): *δ* 164.06, 148.20, 141.17, 133.95, 129.30, 128.85, 127.92, 117.83, 115.47, 115.01, 66.31. Anal. calcd for C_14_H_11_ClN_2_O: C, 65.00; H, 4.29; N, 10.83; found C, 64.98; H, 4.26; N, 10.85.

### 5.5 2-(3-Chlorophenyl)-2,3-dihydroquinazolin-4(1*H*)-one (3e)

White solid; yield: 89%; mp: 184–186 °C; ^1^H NMR (DMSO-*d*_6_, 400 MHz): *δ* 8.38 (s, 1H), 7.58–7.57 (d, *J* = 7.01 Hz, 1H), 7.50 (s, 1H), 7.40–7.37 (m, 3H), 7.24–7.20 (m, 2H), 6.74–6.72 (d, *J* = 7.97 Hz, 1H), 6.67–6.63 (t, *J* = 7.42 Hz, 1H), 5.75 (s, 1H). ^13^C NMR (DMSO-*d*_6_, 100 MHz): *δ* 163.98, 148.05, 144.91, 134.02, 133.51, 130.85, 128.82, 127.91, 127.29, 125.95, 117.87, 115.43, 115.02, 66.08. Anal. calcd for C_14_H_11_ClN_2_O: C, 65.00; H, 4.29; N, 10.83; found C, 65.01; H, 4.33; N, 10.87.

### 5.6 2-(4-Bromophenyl)-2,3-dihydroquinazolin-4(1*H*)-one (3f)

White solid; yield: 87%; mp: 199–201 °C; ^1^H NMR (DMSO-*d*_6_, 400 MHz): *δ* 8.28 (s, 1H), 7.58–7.54 (t, *J* = 7.56 Hz, 3H), 7.42–7.39 (d, *J* = 8.38 Hz, 2H), 7.23–7.19 (t, *J* = 7.56 Hz 1H), 7.09 (s, 1H), 6.72–6.63 (m, 2H), 5.72 (s, 1H). ^13^C NMR (DMSO-*d*_6_, 100 MHz): *δ* 164.00, 148.15, 141.65, 133.93, 131.76, 129.61, 127.89, 122.08, 117.82, 115.47, 114.99, 66.34. Anal. calcd for C_14_H_11_BrN_2_O: C, 55.47; H, 3.66; N, 9.24; found C, 55.48; H, 3.63; N, 9.26.

### 5.7 2-(3-Bromophenyl)-2,3-dihydroquinazolin-4(1*H*)-one (3g)

White solid; yield: 85%; mp: 199–201 °C; ^1^H NMR (DMSO-*d*_6_, 400 MHz): *δ* 8.38 (s, 1H), *δ* 7.64 (s, 1H), 7.58–7.57 (d, *J* = 7.70 Hz, 1H), 7.51–7.45 (m, 2H), 7.33–7.29 (t, *J* = 7.83 Hz, 1H), 7.24–7.20 (m, 2H), 6.74–6.72 (d, *J* = 7.97 Hz, 1H), 6.67–6.63 (t, *J* = 7.83 Hz, 1H), 5.75 (s, 1H). ^13^C NMR (DMSO-*d*_6_, 100 MHz): *δ* 163.98, 148.03, 145.14, 134.03, 131.71, 131.13, 130.18, 127.92, 126.32, 122.15, 117.88, 115.41, 115.02, 66.04. Anal. calcd for C_14_H_11_BrN_2_O: C, 55.47; H, 3.66; N, 9.24; found C, 55.44; H, 3.66; N, 9.23.

### 5.8 2-(4-Methoxyphenyl)-2,3-dihydroquinazolin-4(1*H*)-one (3h)

White solid; yield: 93%; mp: = 181–183 °C; ^1^H NMR (DMSO-*d*_6_, 400 MHz): *δ* 8.17 (s, 1H), 7.57–7.56 (d, *J* = 7.83 Hz, 1H), 7.38–7.36 (d, *J* = 8.79, 2H), 7.21–7.17 (m, 1H), 6.98 (s, 1H), 6.91–6.88 (d, *J* = 8.66 Hz, 2H), 6.70–6.68 (d, *J* = 7.83 Hz, 1H), 6.65–6.61 (m, 1H), 5.66 (s, 1H), 3.69 (s, 3H). ^13^C NMR (DMSO-*d*_6_, 100 MHz): *δ* 164.25, 159.95, 148.56, 133.95, 133.79, 128.76, 127.87, 117.623, 115.51, 114.94, 114.14, 66.83, 55.69. Anal. calcd for C_15_H_14_N_2_O_2_: C, 70.85; H, 5.55; N, 11.02; found C, 70.86; H, 5.55; N, 11.03.

### 5.9 2-(3-Methoxyphenyl)-2,3-dihydroquinazolin-4(1*H*)-one (3i)

Liquid; yield: 92%; ^1^H NMR (DMSO-*d*_6_, 400 MHz): *δ* 8.27 (s, 1H), 7.58–7.56 (d, *J* = 7.70 Hz, 1H), 7.28–7.19 (m, 2H), 7.09 (s, 1H), 7.03 (s, 2H), 6.88–6.86 (d, *J* = 8.11 Hz, 1H), 6.73–6.71 (s, *J* = 8.11 Hz, 1H), 6.65–6.62 (m, 1H), 5.68 (s, 1H), 3.71 (s, 3H). ^13^C NMR (DMSO-*d*_6_, 100 MHz): *δ* 164.09, 159.75, 148.34, 143.86, 133.85, 129.96, 127.87, 119.46, 117.65, 115.51, 114.94, 114.21, 113.10, 66.81, 55.61. Anal. calcd for C_15_H_14_N_2_O_2_: C, 70.85; H, 5.55; N, 11.02; found C, 70.88; H, 5.53; N, 11.01.

### 5.10 2-(Furan-2-yl-phenyl)-2,3-dihydroquinazolin-4(1*H*)-one (3j)

White solid; yield: 92%; mp: 168–170 °C; ^1^H NMR (DMSO-*d*_6_, 400 MHz): *δ* 8.38 (s, 1H), 7.58–7.56 (d, *J* = 7.56 Hz, 2H), 7.22–7.18 (m, 2H), 6.72–6.69 (d, *J* = 7.97 Hz 1H), 6.65–6.62 (t, *J* = 7.70 Hz 2H), 6.34–6.33 (m, 1H), 6.23–6.22 (d, *J* = 3.16 Hz, 1H), 5.71 (s, 1H). ^13^C NMR (DMSO-*d*_6_, 100 MHz): *δ* 163.80, 155.04, 147.66, 143.29, 133.81, 127.79, 117.75, 115.46, 114.99, 110.83, 107.67, 60.71. Anal. calcd for C_12_H_10_N_2_O_2_: C, 67.28; H, 4.71; N, 13.08; found C, 67.29; H, 4.69; N, 13.09.

### 5.11 2-(2-Nitrophenyl)-2,3-dihydroquinazolin-4(1*H*)-one (3k)

Yellow solid; yield: 87%; ^1^H NMR (DMSO-*d*_6_, 400 MHz): *δ* 8.18 (s, 1H), 7.95 (s, 1H), 7.78–7.48 (m, 2H), 7.15 (s, 2H), 6.93 (s, 2H), 6.70–6.62 (d, *J* = 34.90 Hz, 2H), 6.29 (s, 1H). ^13^C NMR (DMSO-*d*_6_, 100 MHz): *δ* 163.63, 147.72, 147.24, 136.07, 134.22, 133.99, 133.71, 129.93, 129.02, 127.47, 124.84, 117.83, 115.04, 114.60, 62.34. Anal. calcd for C_14_H_11_N_3_O_3_: C, 62.45; H, 4.12; N, 15.61; found C, 62.47.; H, 4.11; N, 15.64.

### 5.12 2-(3-Nitrophenyl)-2,3-dihydroquinazolin-4(1*H*)-one (3l)

Yellow solid; yield: 89%; ^1^H NMR (DMSO-*d*_6_, 400 MHz): *δ* 8.59 (s, 1H), 8.38 (s, 1H), 8.19–8.17 (d, *J* = 8.11 Hz, 1H), 7.96–7.94 (d, *J* = 7.70 Hz, 1H), 7.68–7.63 (m, 2H), 7.56–7.54 (d, *J* = 7.83 Hz, 1H), 7.37 (s, 1H), 7.28–7.24 (t, *J* = 7.28 Hz, 1H), 7.14–7.10 (t, *J* = 7.28 Hz, 1H), 5.97 (s, 1H). ^13^C NMR (DMSO-*d*_6_, 100 MHz): *δ* 163.58, 147.78, 147.40, 133.73, 133.44, 132.02, 130.07, 128.87, 117.68, 116.54, 115.01, 114.71, 114.52, 65.32. Anal. calcd for C_14_H_11_N_3_O_3_: C, 62.45; H, 4.12; N, 15.61; found C, 62.44.; H, 4.12; N, 15.62.

## Author contributions

S. K., G. R., S. H., and R. C. designed the schemes. S. K. performed the experiments. S. K., and G. R. evaluated the data and prepared the figures and tables. G. R., S. K., S. H., and R. C. revised and reviewed the manuscript.

## Conflicts of interest

The authors declare no competing financial interest.

## Supplementary Material

RA-013-D2RA07496F-s001
