# Supplementary material for: An efficient approach for the green synthesis of biologically active 2,3-dihydroquinazolin-4(1H)-ones using a magnetic EDTA coated copper based nanocomposite
Source: RSC Adv. 2023 Jan 11;13(3):1923–32. doi: 10.1039/d2ra07496f (PMC9832363; doi:10.1039/d2ra07496f)
Supplement: RA-013-D2RA07496F-s001 [file RA-013-D2RA07496F-s001.pdf]

Electronic Supplementary Material (ESI) for RSC Advances.
This journal is © The Royal Society of Chemistry 2022

An efficient approach for the green synthesis of biologically active 2,3-dihydroquinazolin-4(1H)-ones using EDTA coated magnetic copper nanocatalyst

Sahil Kohli,[1,2] Garima Rathee,[1] Sunita Hooda[2,a*] and Ramesh Chandra[1,3,4,b*]

1- Drug Discovery & Development Laboratory, Department of Chemistry, University of Delhi, Delhi-110007, India.
2- Department of Chemistry, Acharya Narendra Dev College, University of Delhi, Delhi-110019, India.
3- Dr. B.R. Ambedkar Center for Biomedical Research (ACBR), University of Delhi, Delhi-110007, India.
4- Institute of Nanomedical Science (INMS), University of Delhi, Delhi-110007, India.

***Correspondence:**

(a) Dr. Sunita Hooda, Department of Chemistry, Acharya Narendra Dev College, University of Delhi, Delhi-110019, India. E-mail: sunitahooda@andc.du.ac.in

(b) Prof. Ramesh Chandra, Drug Discovery and Development Laboratory, Department of Chemistry, University of Delhi, Delhi-110007, India. E-mail: rameshchandragroup@gmail.com.

SUPPLEMENTARY INFORMATION (SI)

Supplementary Information (SI) includes XRD, SEM and TEM of recycled Fe_3O_4@EDTA/CuI nanocatalyst; Green chemistry metric calculations, ^1H NMR and ^{13}C NMR spectra of all compounds.

Total No of Pages: 18, Total No of Tables: 0, Total number of Figures: 3.

Table of contents

S.No.	Content	Page number
1.	General Remarks	S3
2.	Characterization of recycled Fe_3O_4@EDTA/CuI nanocatalyst (XRD, SEM, and TEM)	S4
3.	Calculation of Green chemistry metrices	S5-S6
4.	^1H and ^{13}C spectra of compounds	S7-S18

1. General Remarks

Chemicals and solvents were purchased from Sigma Adrich, Alfa-Aesar and Merck India Pvt. ^{1}H and ^{13}C spectra were recorded on a Jeol Spectrospin spectrometer at 400 MHz and 100 MHz respectively by keeping TMS as internal standard. CHNS Analyser was recorded on model Vario Micro Cube at USIC (University Science Instrument Centre), University of Delhi, Delhi, India. The X-ray diffractometer (Model No. D8 DISCOVER) at 2θ range of 2–90° with Cu Kα radiation. Chemical shift values were recorded in terms of δ and coupling constants (J) are in hertz (Hz). FTIR spectra were obtained on IRAffinity-1S Fourier Transform Infrared Spectrophotometer. Scanning Electron microscopy (SEM) measurement was performed on JEOL JSM 6610 at USIC, University of Delhi. Transmission electron microscopy (TEM) was obtained on a TECNAI G20 HR-TEM 200kV at SAIF (sophisticated analytical instrumentation facility), AIIMS, New Delhi, India. The elemental composition and electronic structure analysis were obtained from X-ray photoelectron spectra (XPS) of PHI 5000 Versa Probe III instrument at Institute Instrumentation Centre, Indian Institute of Technology, Roorkee – 247 667 (Uttarakhand), India. ICP-MS was measured on Agilent ICP-MS 7900 with UHMI at the ICP-MS Lab, (CRF Adopted Facility), IIT Delhi, INDIA.

2. Characterization of recycled Fe_3O_4@EDTA/CuI nanocatalyst

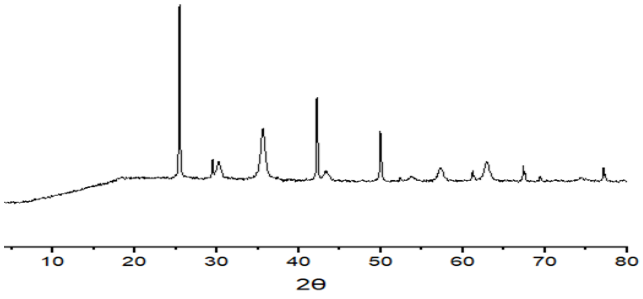

Figure S1: XRD of recycled catalyst

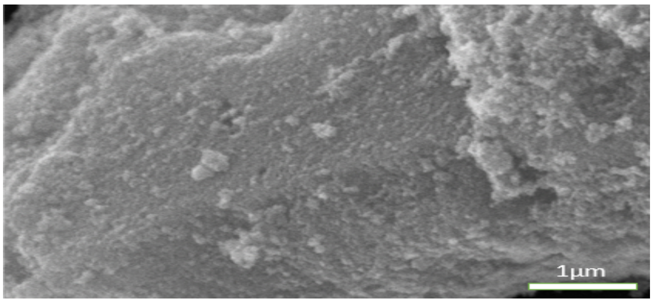

Figure S2: SEM image of recycled catalyst

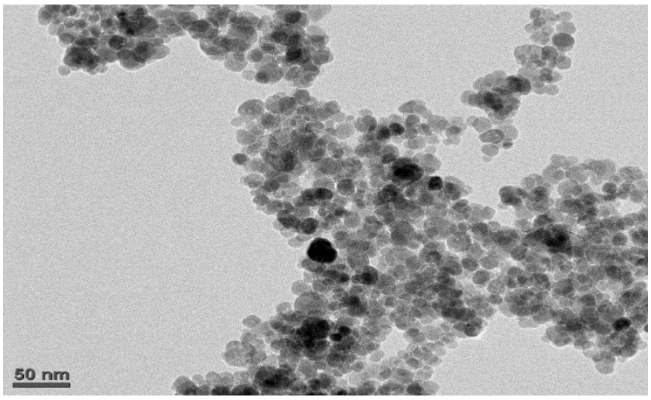

Figure S3: TEM image of recycled catalyst

3. Calculation of Green chemistry metrices

Calculation of Green chemistry metrices for 3c of our method

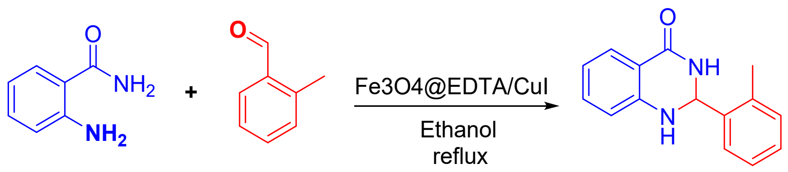

FW	136.15 g/mol	120.15 g/mol	238.29 g/mol
**mmol**	**0.5 mmol**	**0.5 mmol**	**0.48 mmol**
**amount**	**68 mg**	**60 mg**	**116 mg**

E-factor:

The ideal value of E-factor is zero.

E-factor = [total mass of raw materials - the total mass of product]/ mass of product.

E-factor of i = [(68 + 60)-116]/116

= 0.10.

Process mass intensity (PMI):

PMI= ∑ (mass of stoichiometric reactants)/[mass of product]

= (68+ 60)/ 116

= 1.10

Reaction mass efficiency (RME):

RME = [mass of product /∑ (mass of stoichiometric reactants)] × 100

= [116/ (68 + 60)] × 100

= 90.62%

Carbon efficiency (CE):

CE= [Amount of carbon in product/ Total carbon present in reactants] x 100

= [no. of moles of product x no. of carbons in product / (moles of a x carbons in 1i + moles of 2 carbons in 2)] x 100

= [0.48 x 15 / (0.5 x 7 + 0.5 x 8)] x 100

= [7.2 / (3.5 + 4)] x 100

= 96%

Calculation of Green chemistry metrices for previous reported method[1]

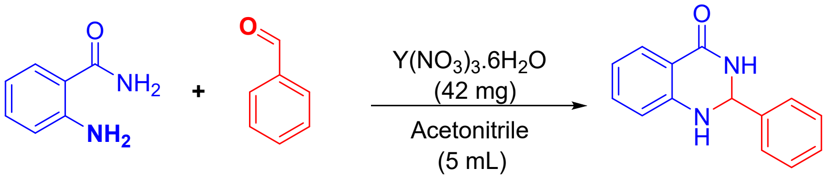

**FW**	136.15 g/mol	106.12 g/mol	224 g/mol
**mmol**	**1.1 mmol**	**1.2 mmol**	**1.08 mmol**
**amount**	**150 mg**	**128 mg**	**242 mg**

E-factor:

The ideal value of E-factor is zero.

E-factor = [total mass of raw materials - the total mass of product]/ mass of product.

E-factor of i = [(42 + 150 + 128 + 3930)-242]/242

= 16.56.

Process mass intensity (PMI):

PMI= ∑ (mass of stoichiometric reactants + solvent)/[mass of product]

= (42 + 150 + 128 + 3930)/ 242

= 17.56

Reaction mass efficiency (RME):

RME = [mass of product /∑ (mass of stoichiometric reactants)] × 100

= [242/ (42 + 150 + 128)] × 100

= 75%

Carbon efficiency (CE):

CE= [Amount of carbon in product/ Total carbon present in reactants] x 100

= [no. of moles of product x no. of carbons in product / (moles of a x carbons in 1i + moles of 2 carbons in 2)] x 100

= [1.08 x 14 / (1.1 x 7 + 1.2 x 7)] x 100

= 93%

Reference

1. A.A. Khan, K. Mitra, A. Mandal, N. Baildya, M.A. Mondal, *Heteroat. Chem.*, 2017, ***28***, 21379

^{1}H and ^{13}C NMR

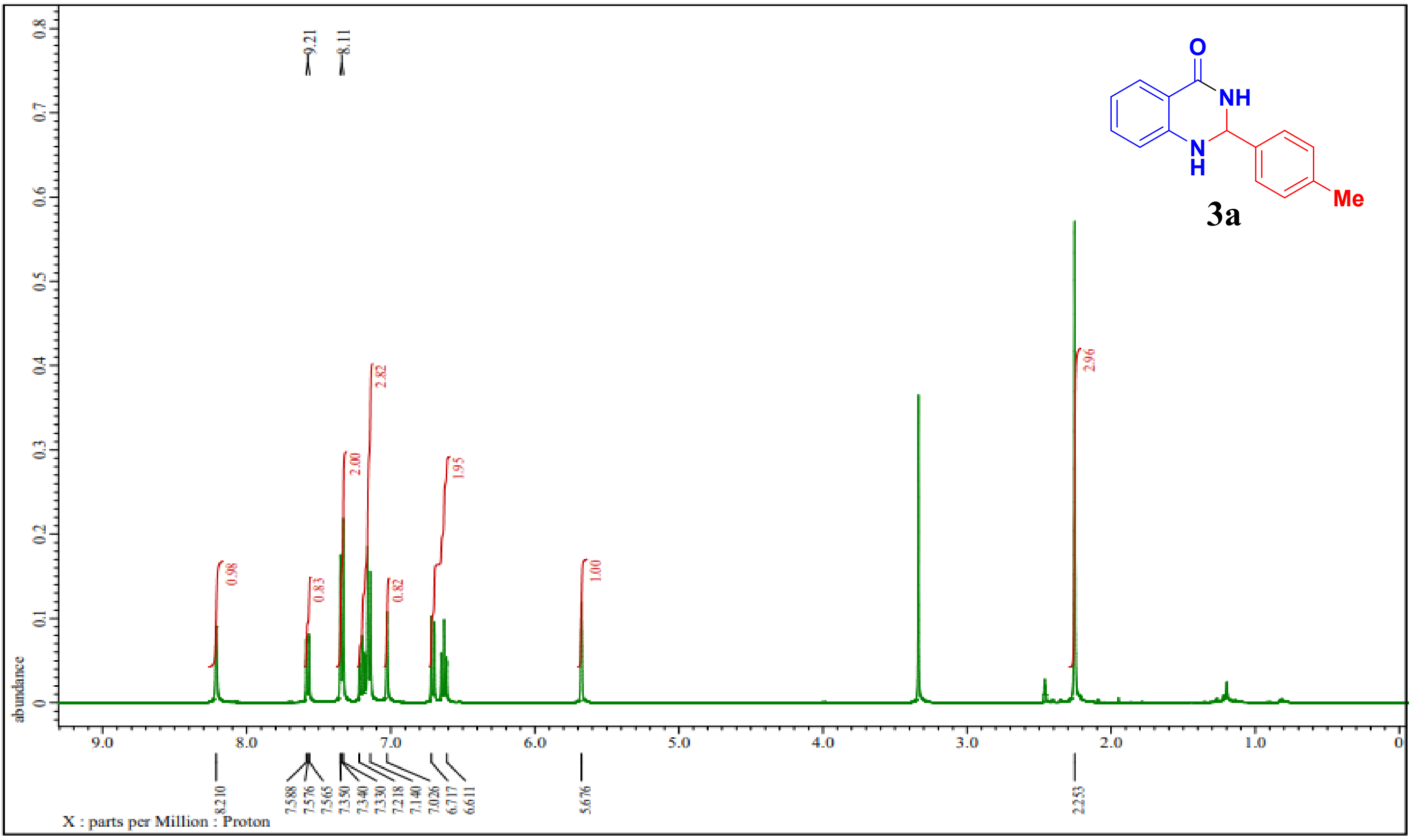

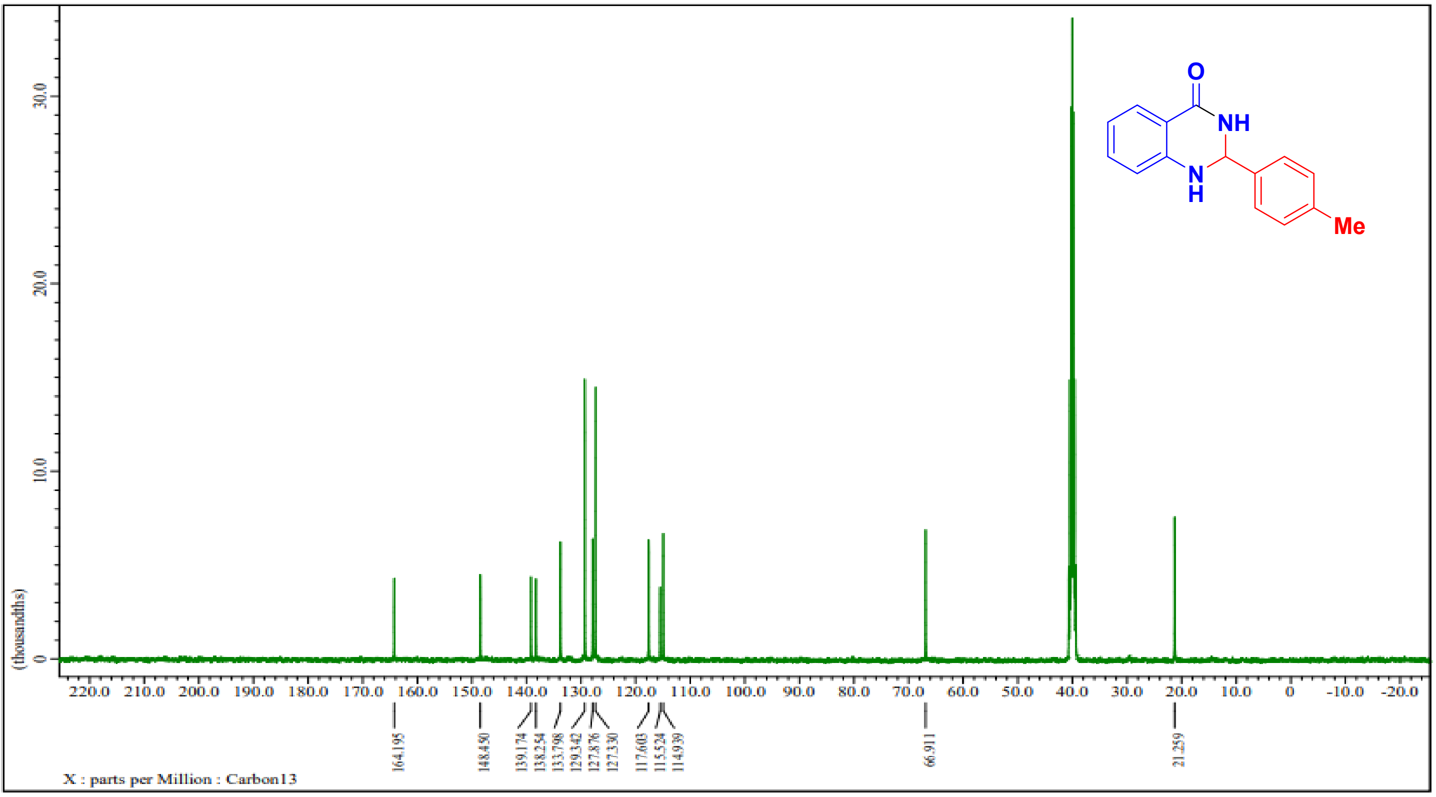

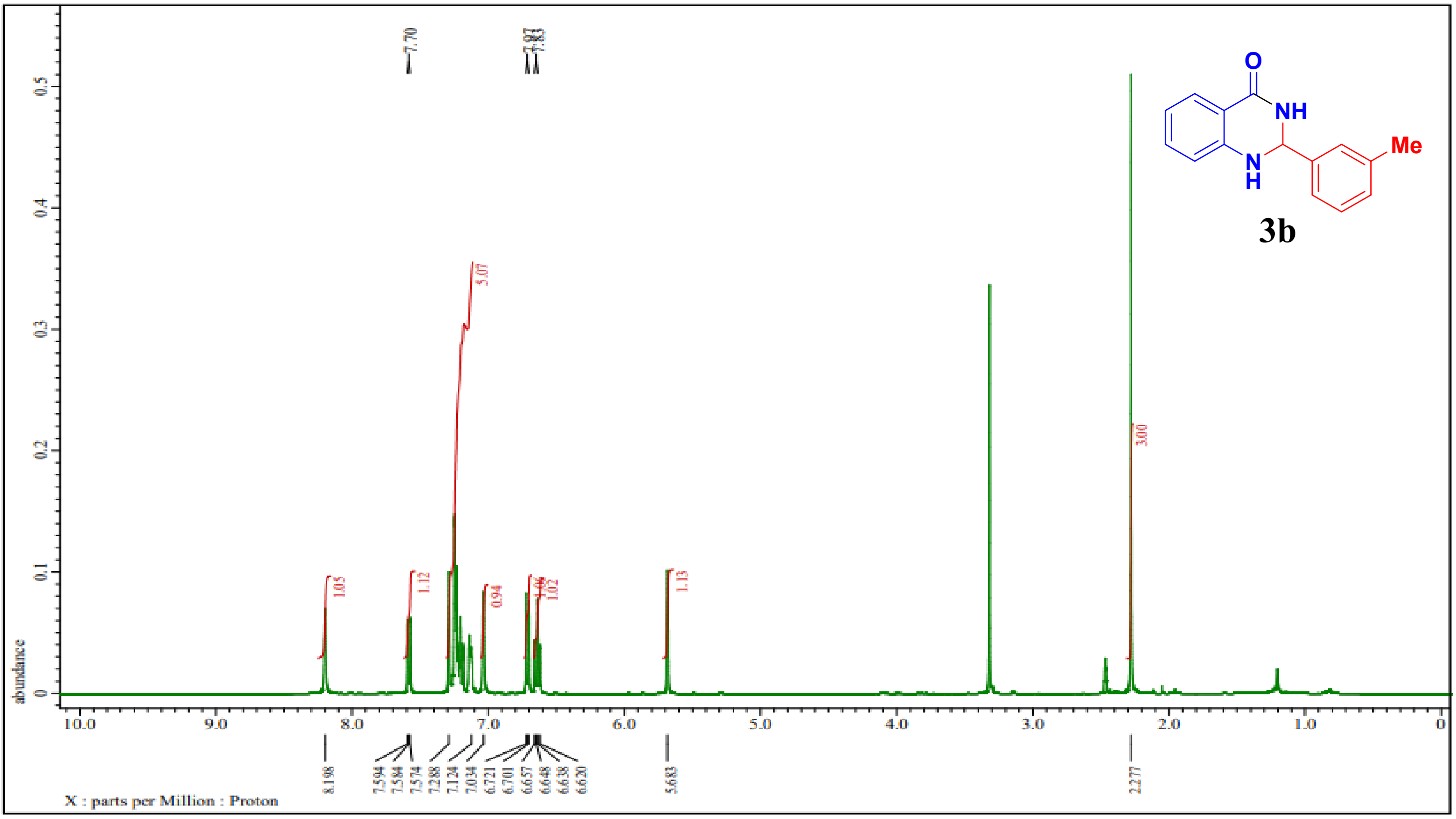

3b
X : parts per Million : Proton
abundance

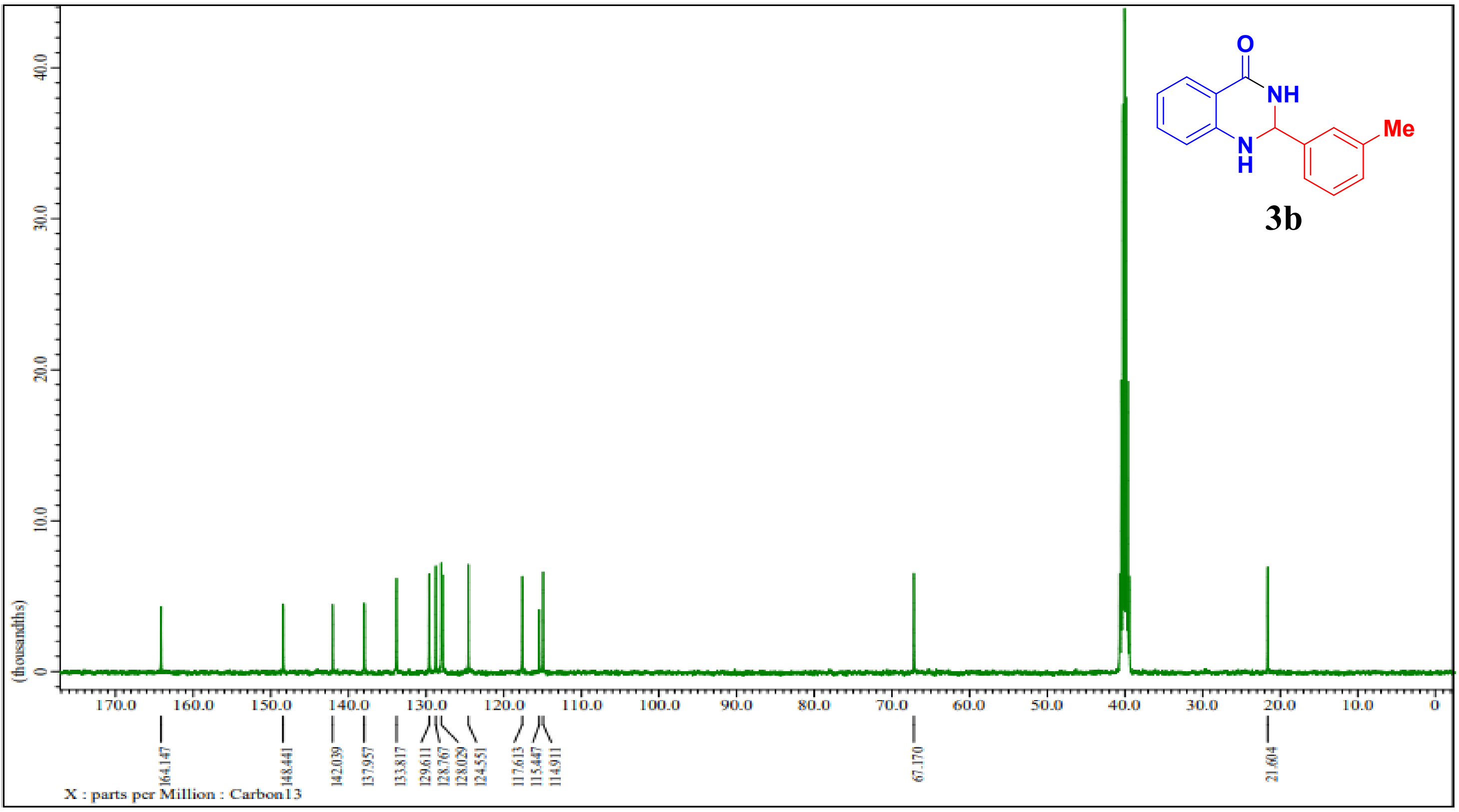

3b
X : parts per Million : Carbon13
(thousandths)

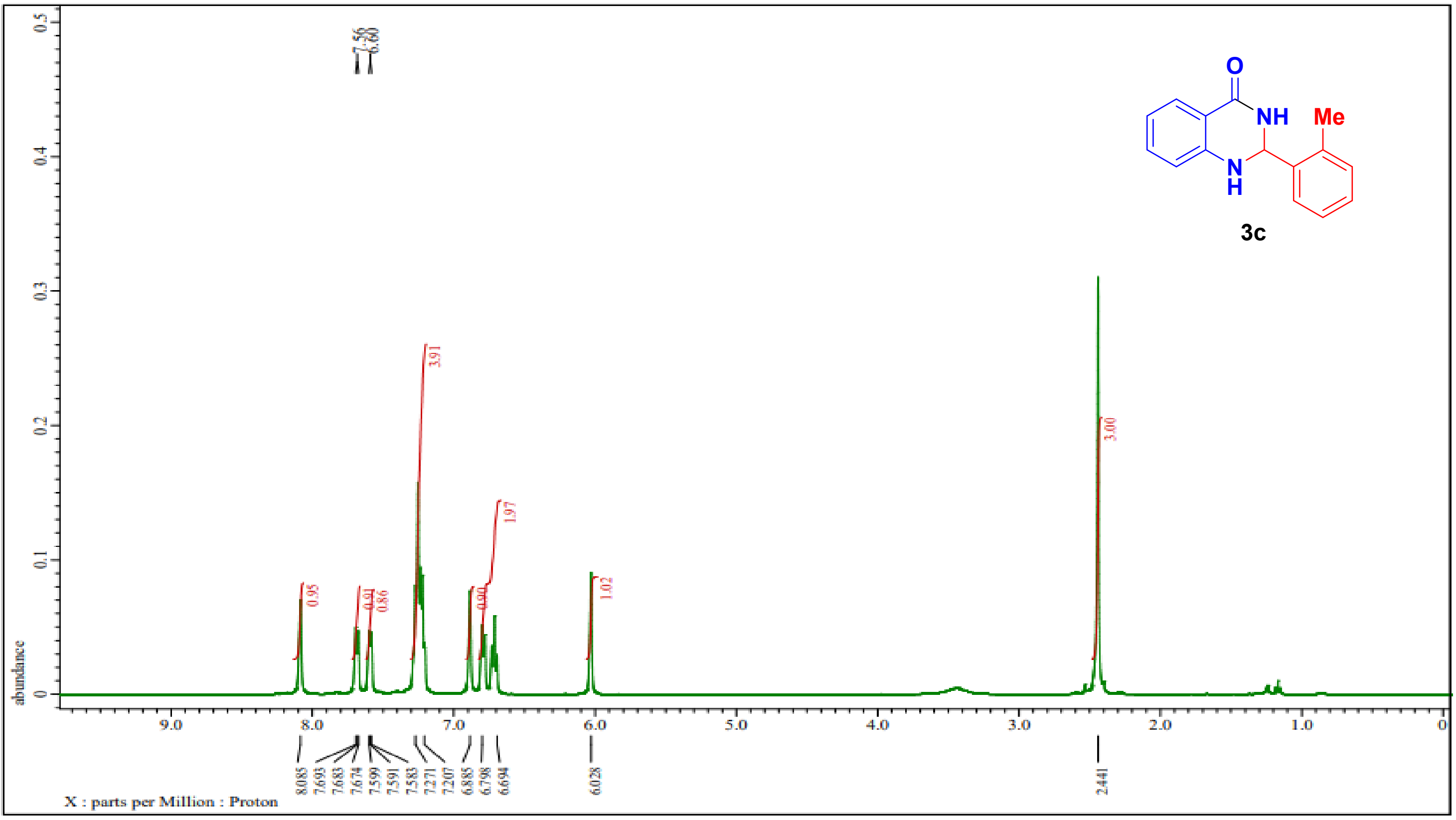
3c
X : parts per Million : Proton
abundance

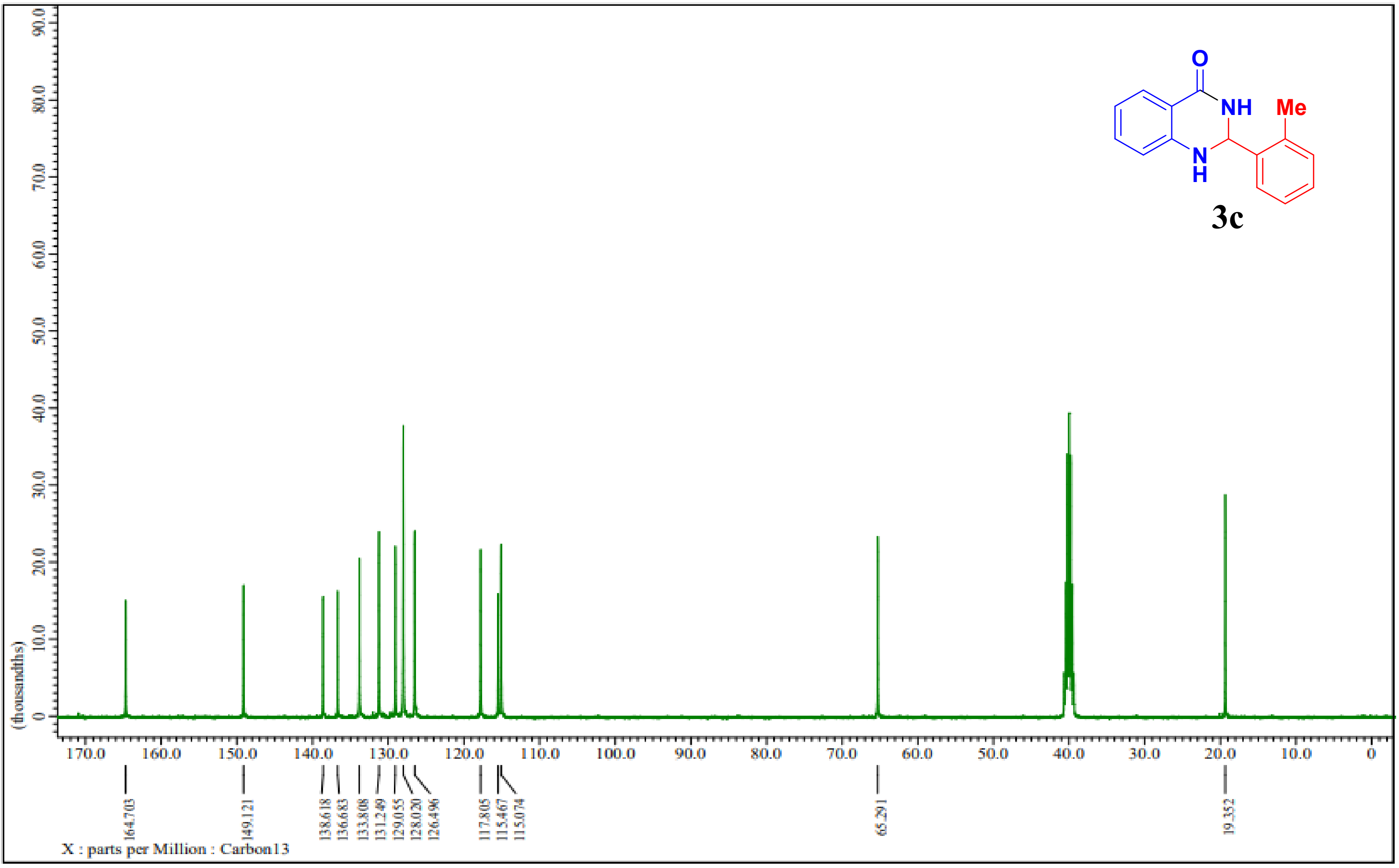
3c
X : parts per Million : Carbon13
(thousandths)

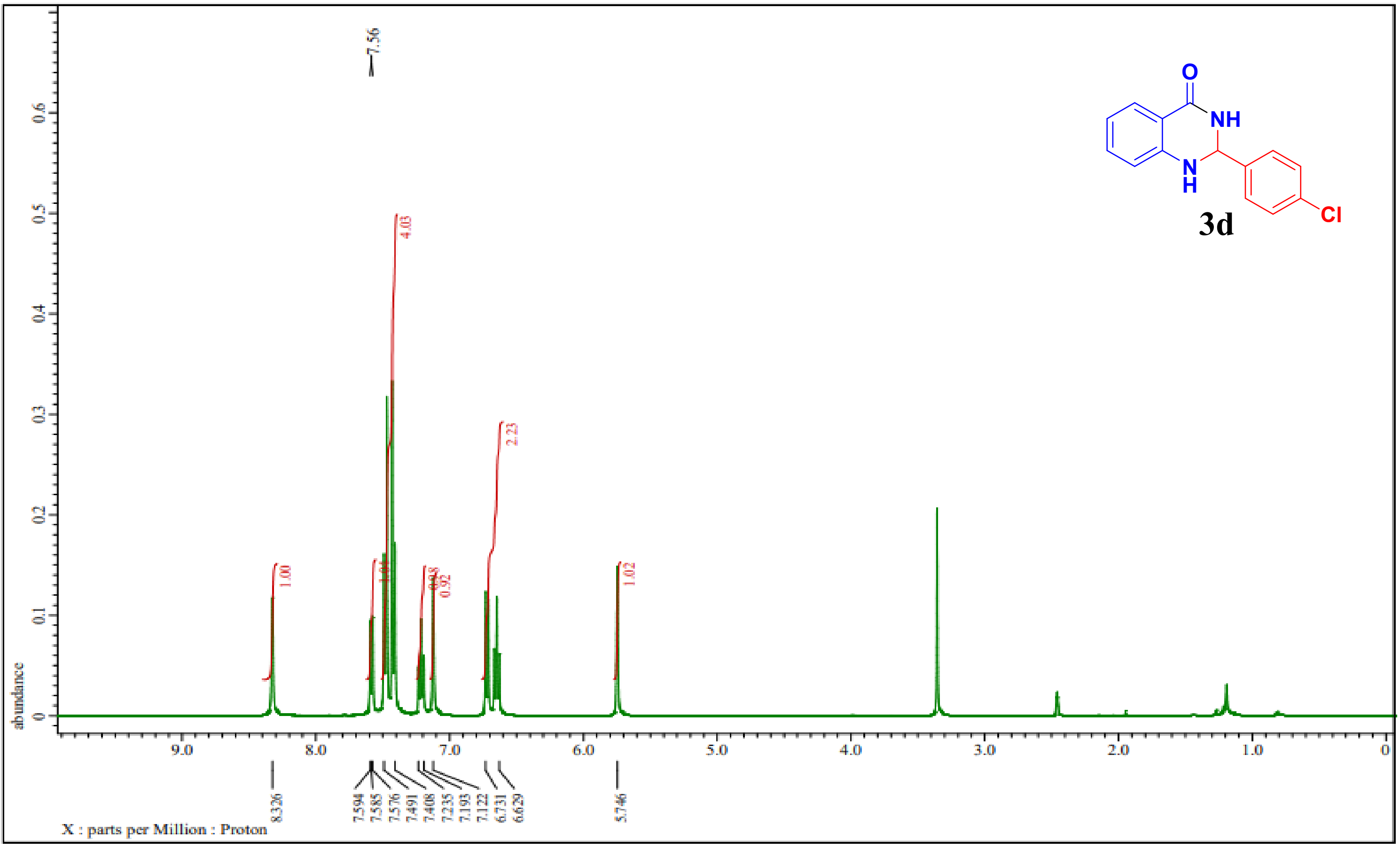
7.56
1.00
4.03
2.23
0.92
1.02
abundance
X : parts per Million : Proton
8.326
7.594
7.585
7.576
7.491
7.408
7.235
7.193
7.122
6.731
6.629
5.746
O
NH
N
H
Cl
3d

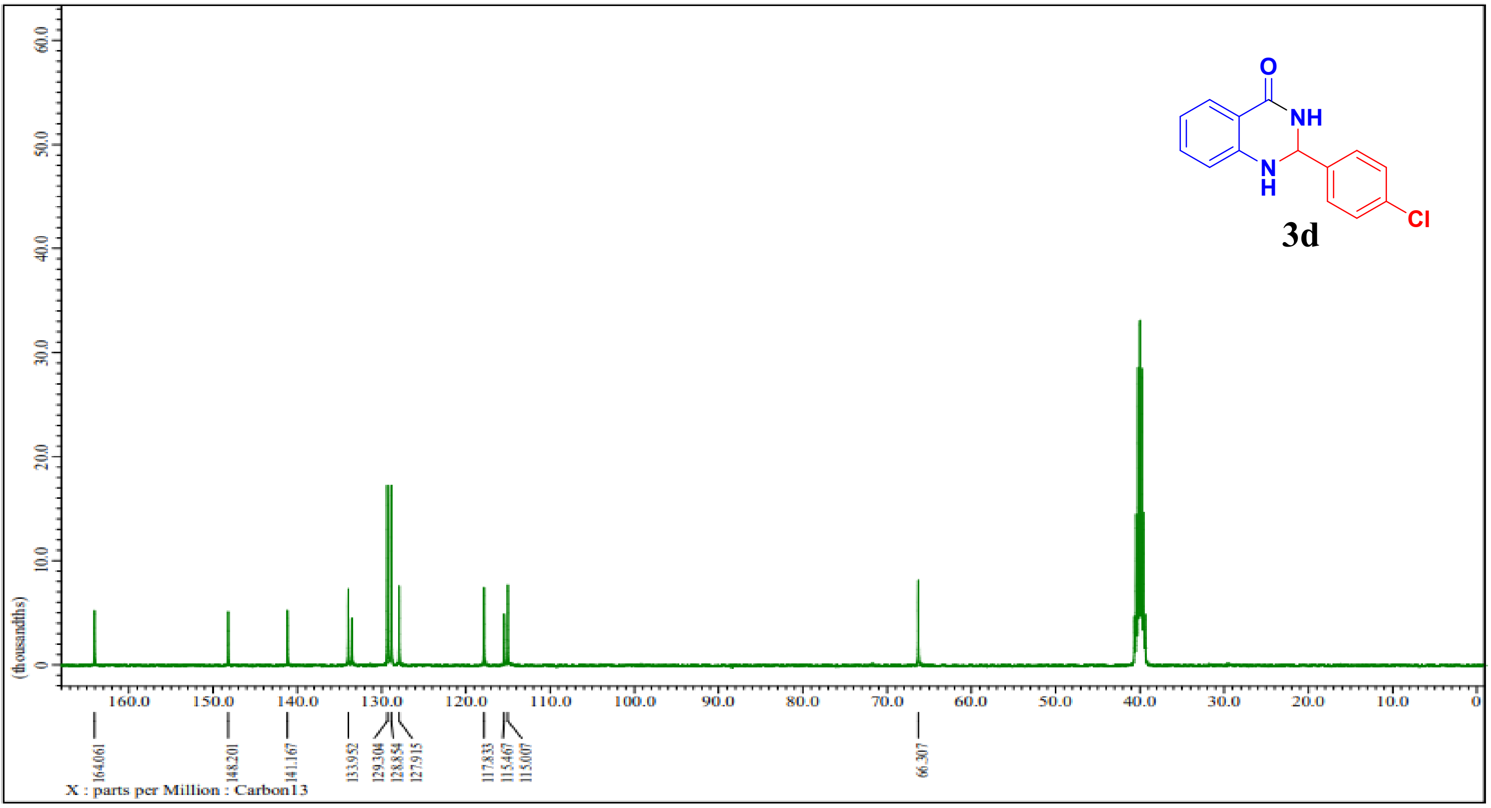
(thousandths)
X : parts per Million : Carbon13
164.061
148.201
141.167
133.952
129.304
128.854
127.915
117.833
115.467
115.007
66.307
O
NH
N
H
Cl
3d

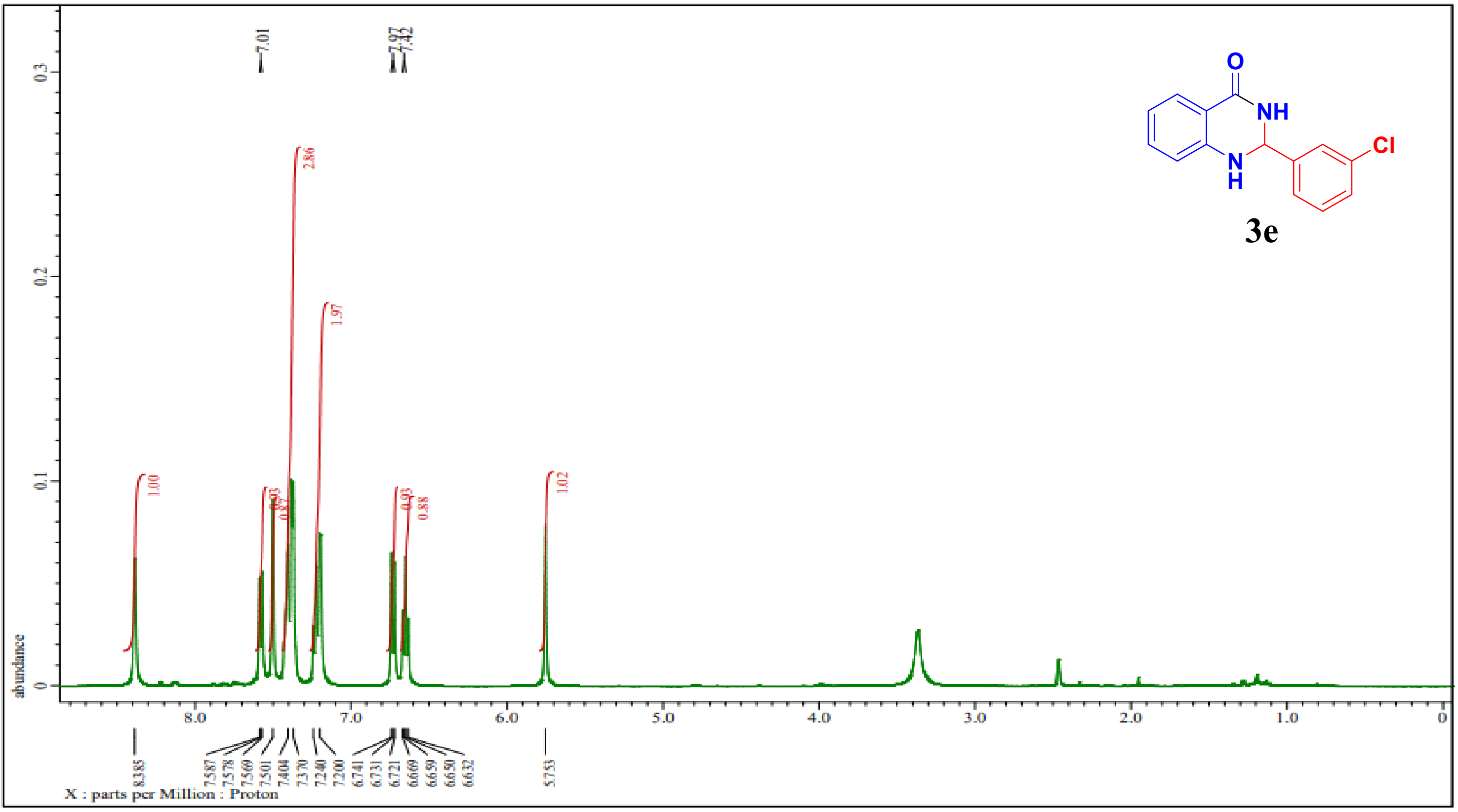
O
NH
N
H
Cl
3e
abundance
X : parts per Million : Proton

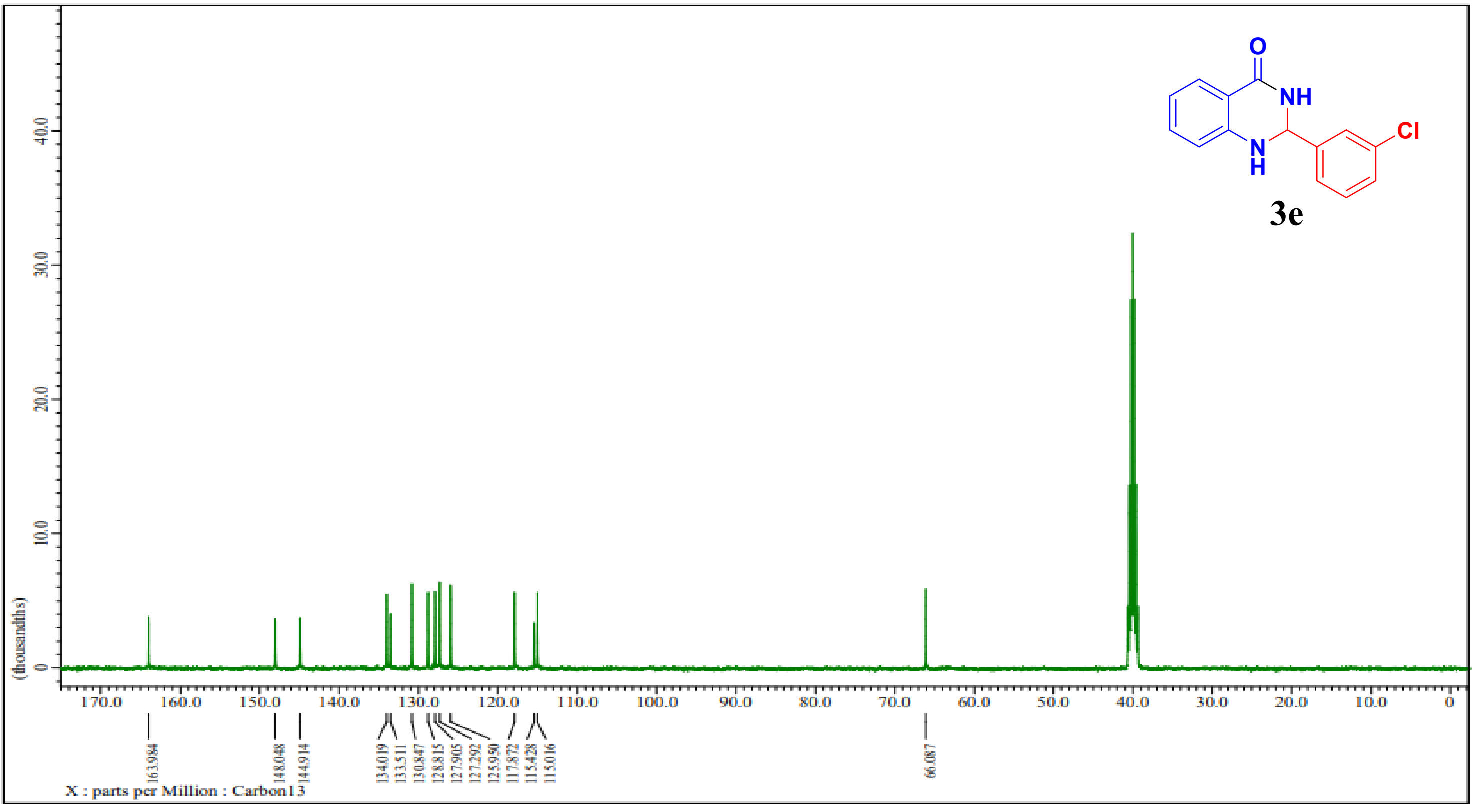
O
NH
N
H
Cl
3e
(thousandths)
X : parts per Million : Carbon13

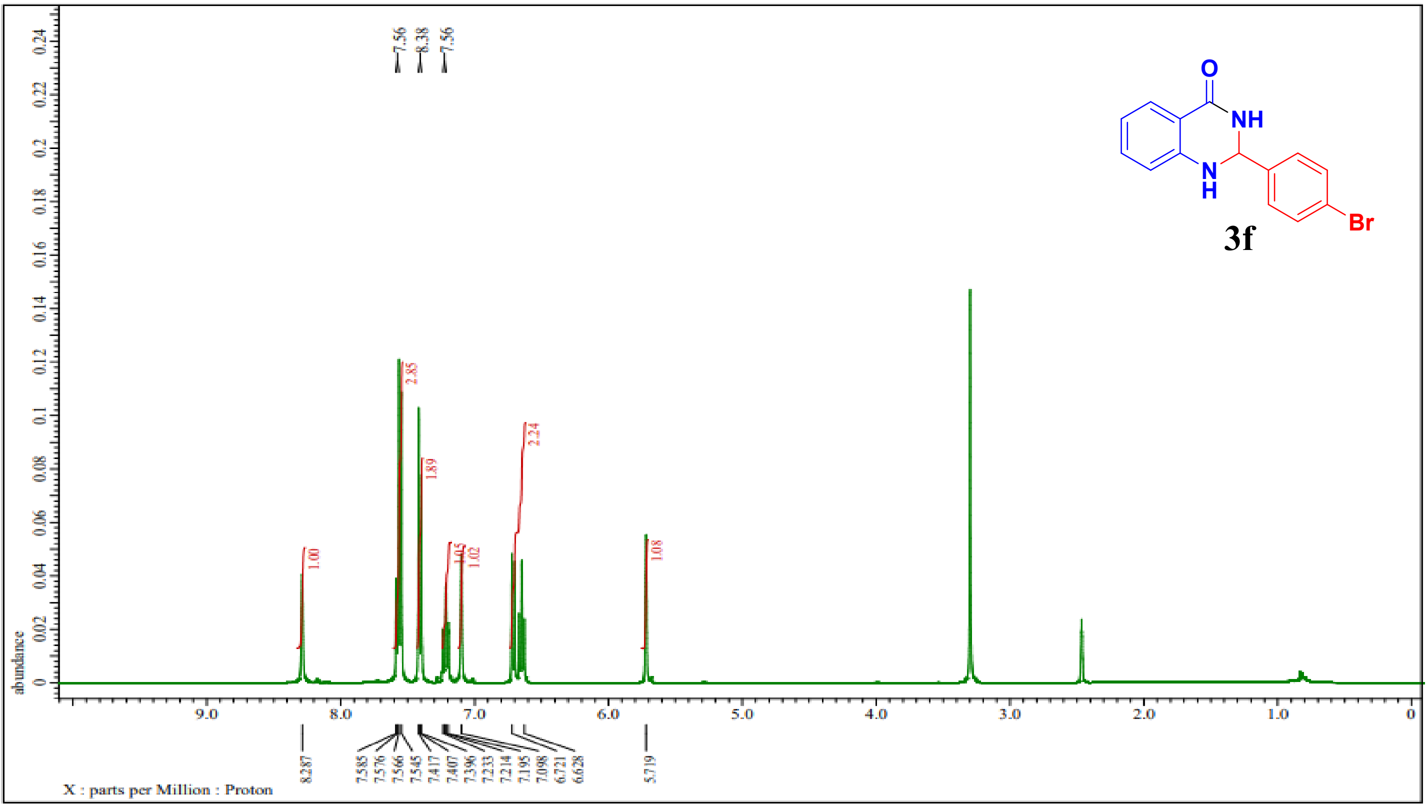

3f
O
NH
N
H
Br
7.56
8.38
7.56
1.00
2.85
1.89
1.05
1.02
2.24
1.08
abundance
X : parts per Million : Proton
8.287
7.585
7.576
7.566
7.545
7.417
7.407
7.396
7.233
7.214
7.195
7.098
6.721
6.628
5.719

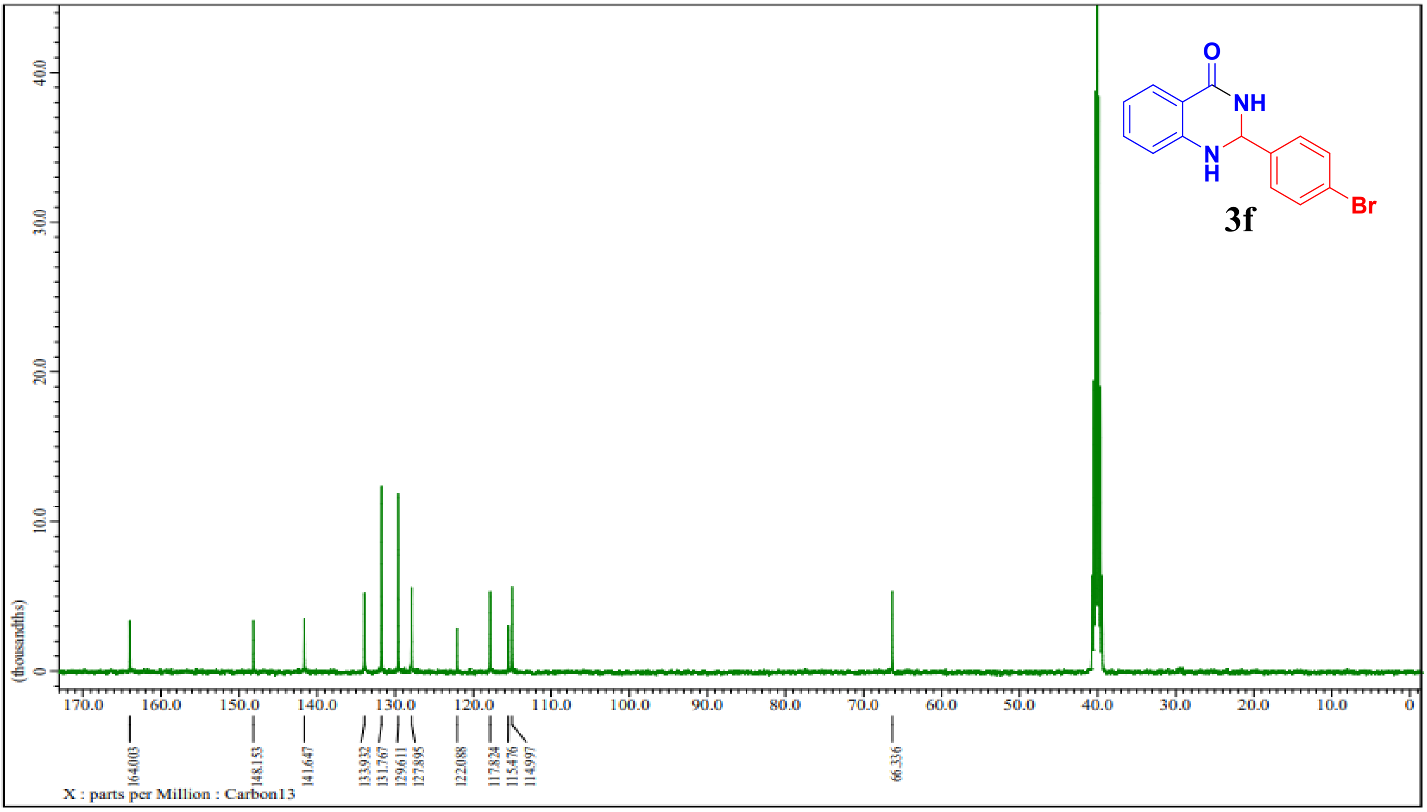

3f
O
NH
N
H
Br
(thousandths)
X : parts per Million : Carbon13
164.003
148.153
141.647
133.932
131.767
129.611
127.895
122.088
117.824
115.476
114.997
66.336

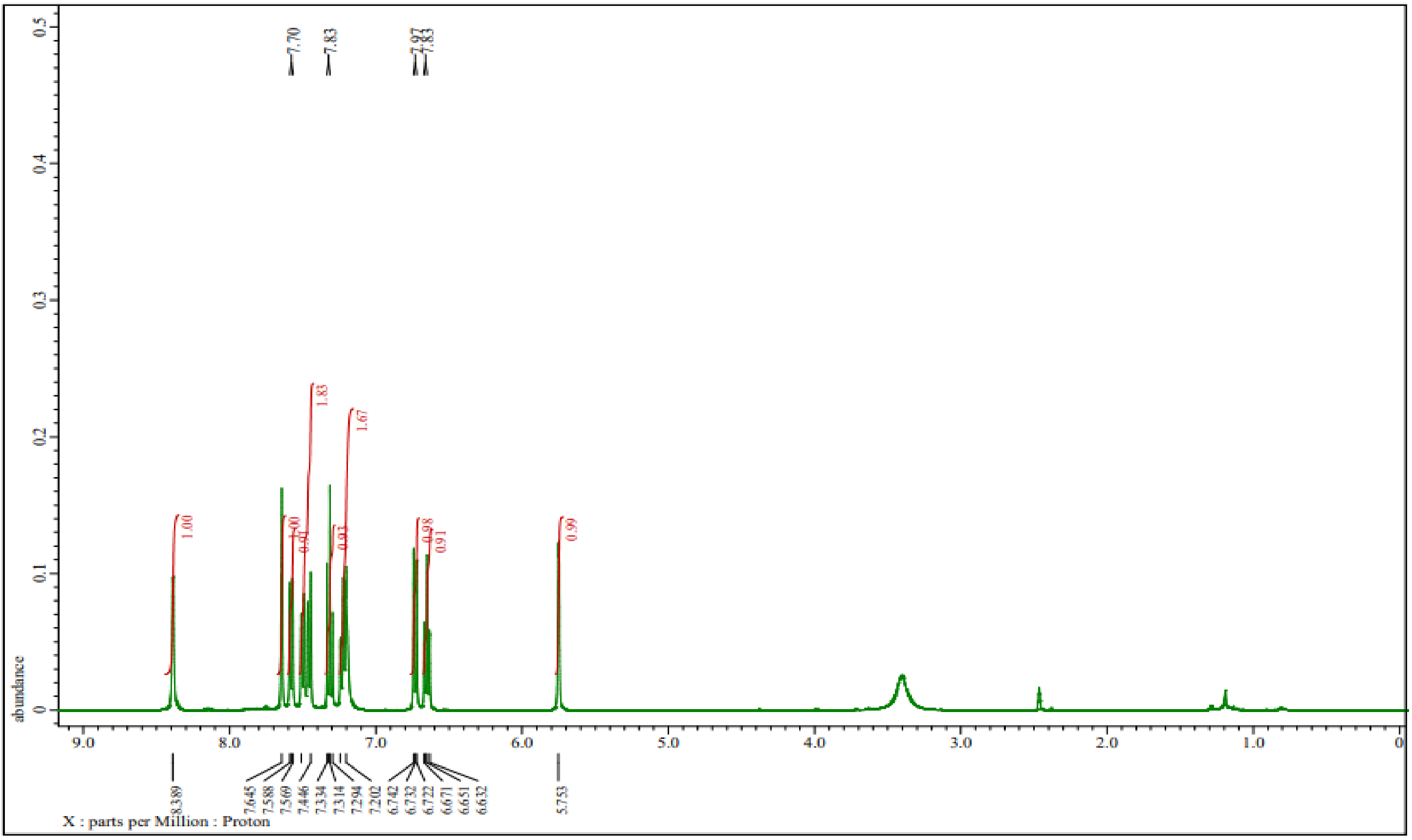
abundance
X : parts per Million : Proton
8.389
7.645
7.588
7.569
7.446
7.334
7.314
7.294
7.202
6.742
6.732
6.722
6.671
6.651
6.632
5.753

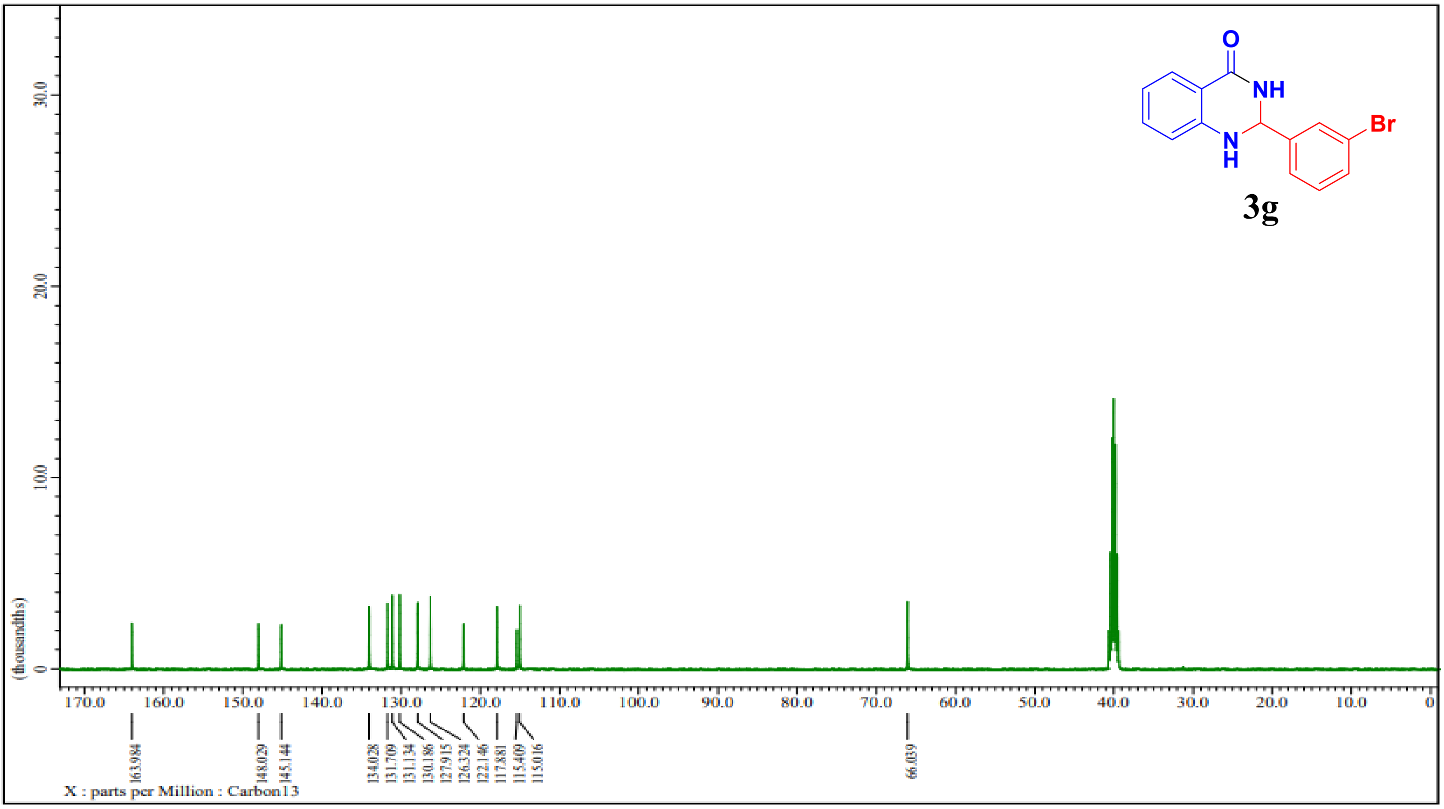
(thousandths)
X : parts per Million : Carbon13
163.984
148.029
145.144
134.028
131.709
131.134
130.186
127.915
126.324
122.146
117.881
115.409
115.016
66.039
O
NH
Br
N
H
3g

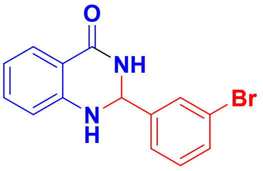
O
NH
Br
N
H

3g

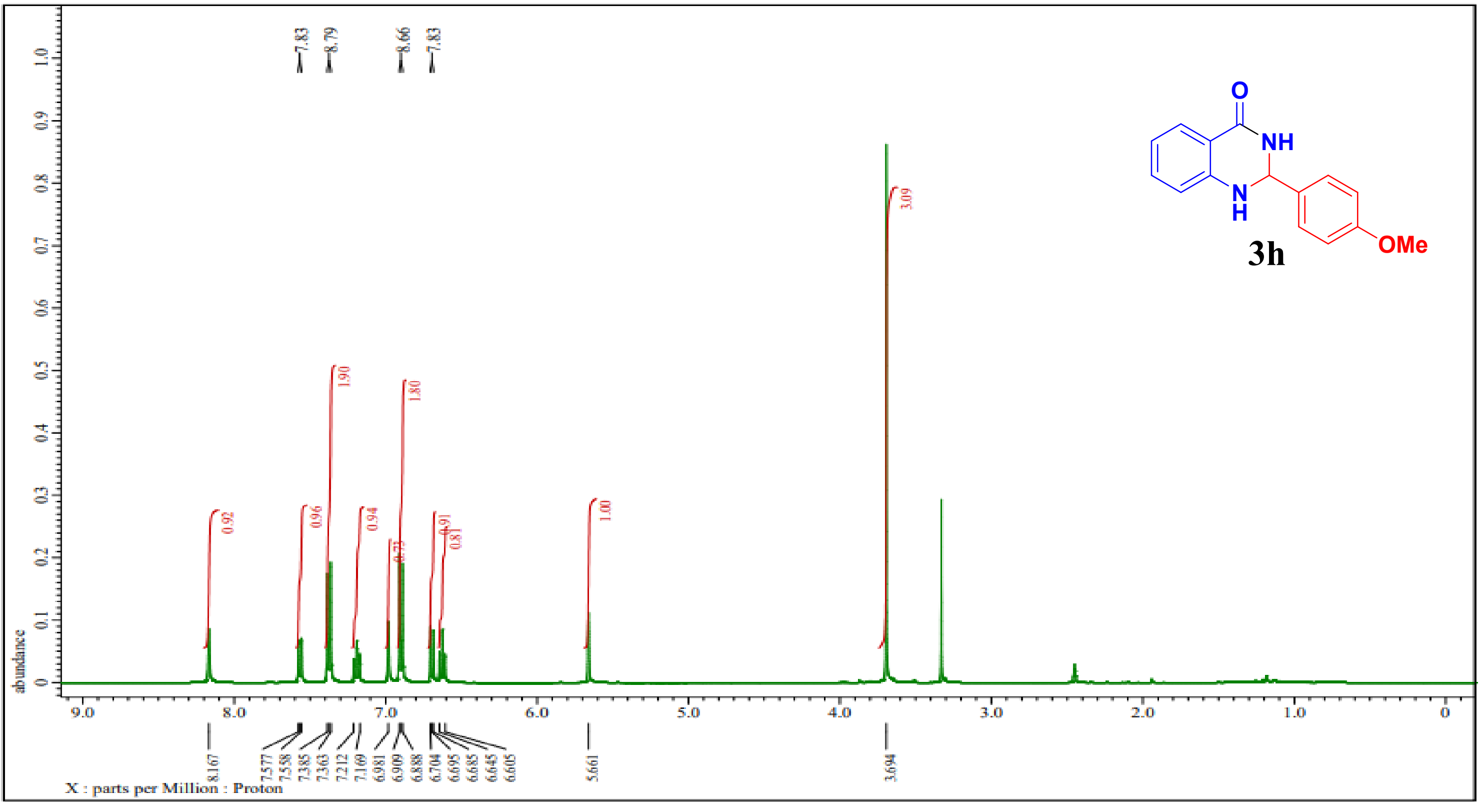
3h
O
NH
N
H
OMe
abundance
X : parts per Million : Proton
8.167
7.577
7.558
7.385
7.363
7.212
7.169
6.981
6.909
6.888
6.704
6.695
6.685
6.645
6.605
5.661
3.694
0.92
0.96
1.90
0.94
1.80
1.00
3.09

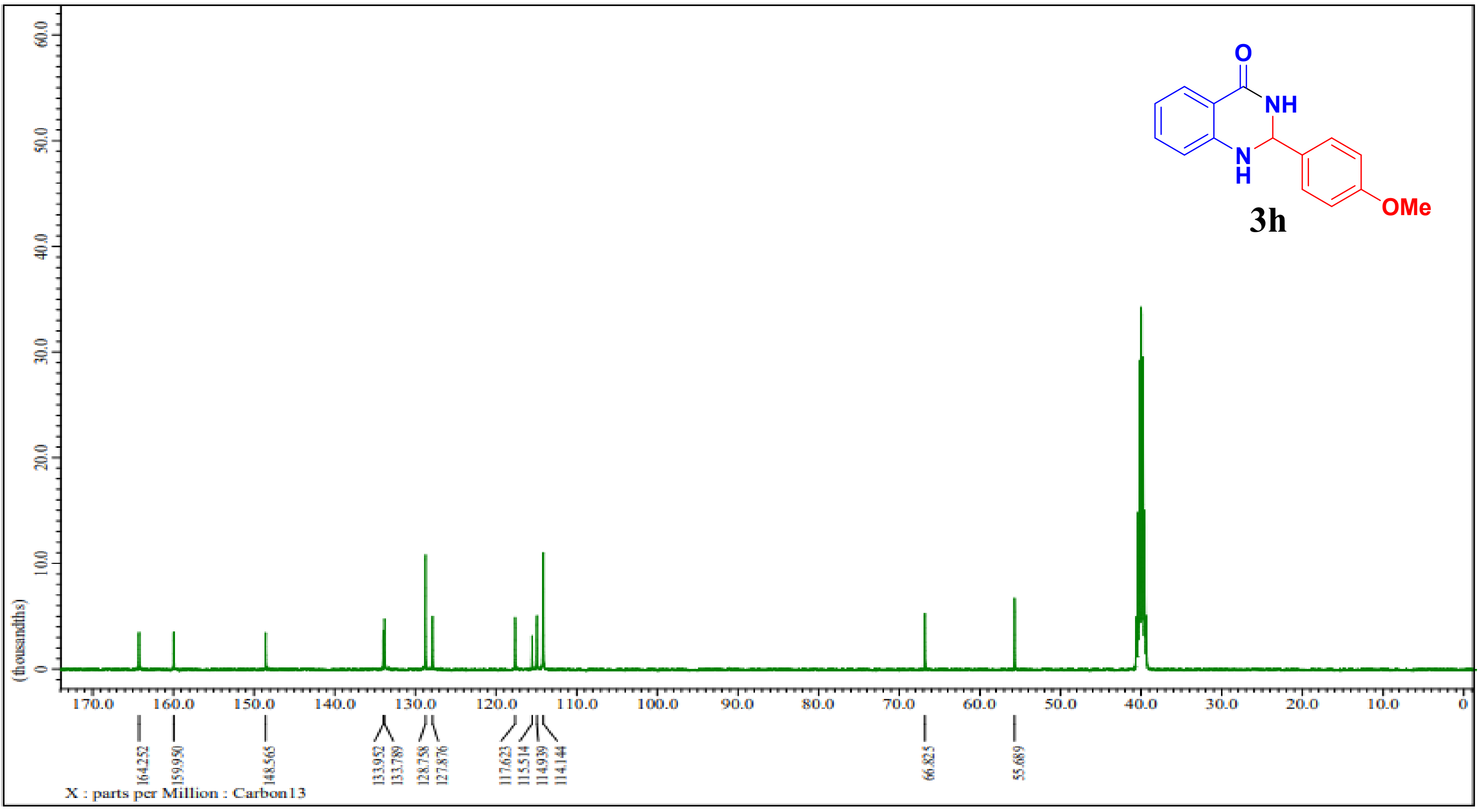
3h
O
NH
N
H
OMe
(thousandths)
X : parts per Million : Carbon13
164.252
159.950
148.565
133.952
133.789
128.758
127.876
117.623
115.514
114.939
114.144
66.823
55.689

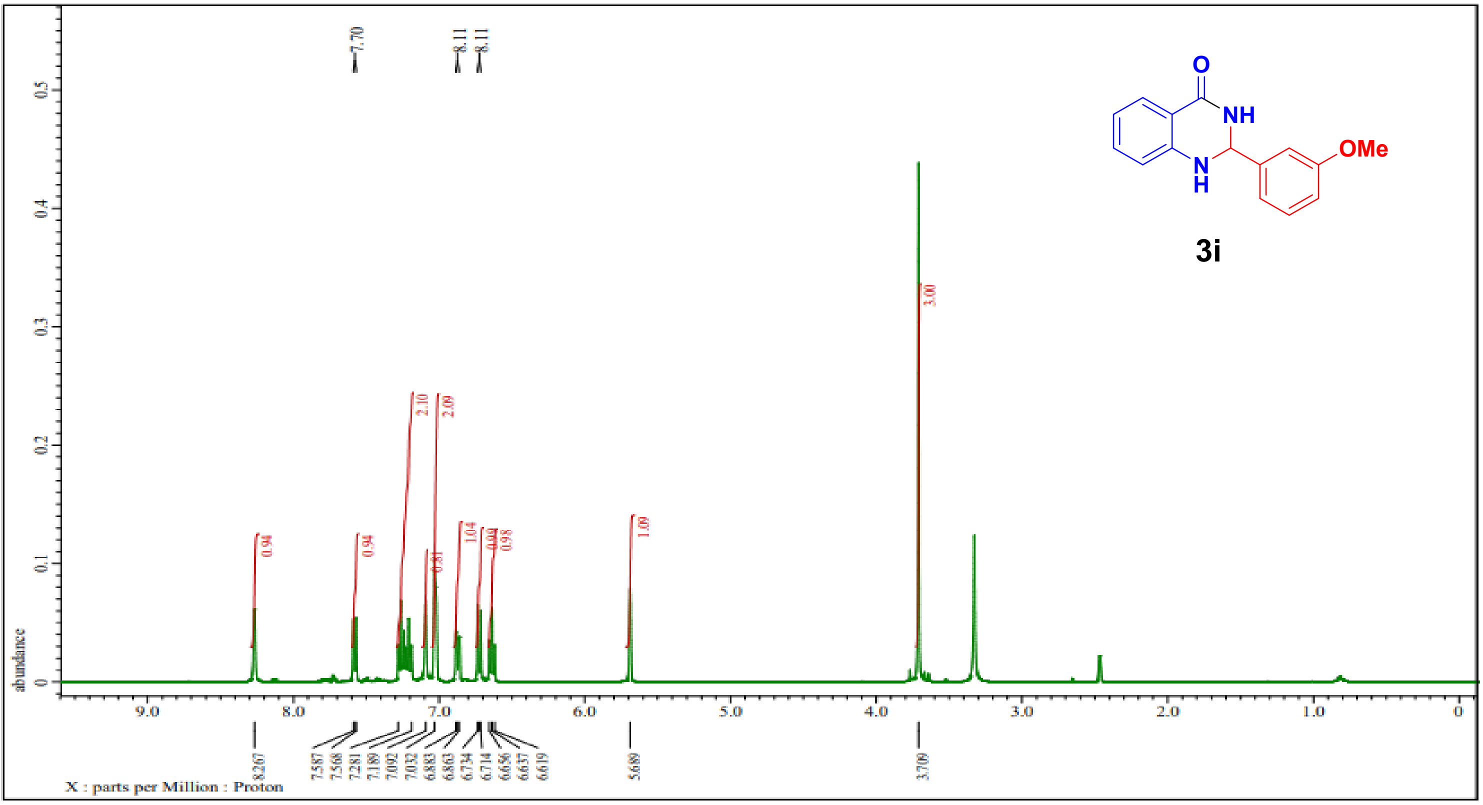
O
NH
N
H
OMe
3i
abundance
7.70
8.11
8.11
0.94
0.94
2.10
2.09
1.04
0.98
1.09
3.00
8.267
7.587
7.568
7.281
7.189
7.092
7.032
6.883
6.863
6.734
6.714
6.656
6.637
6.619
5.689
3.709
X : parts per Million : Proton

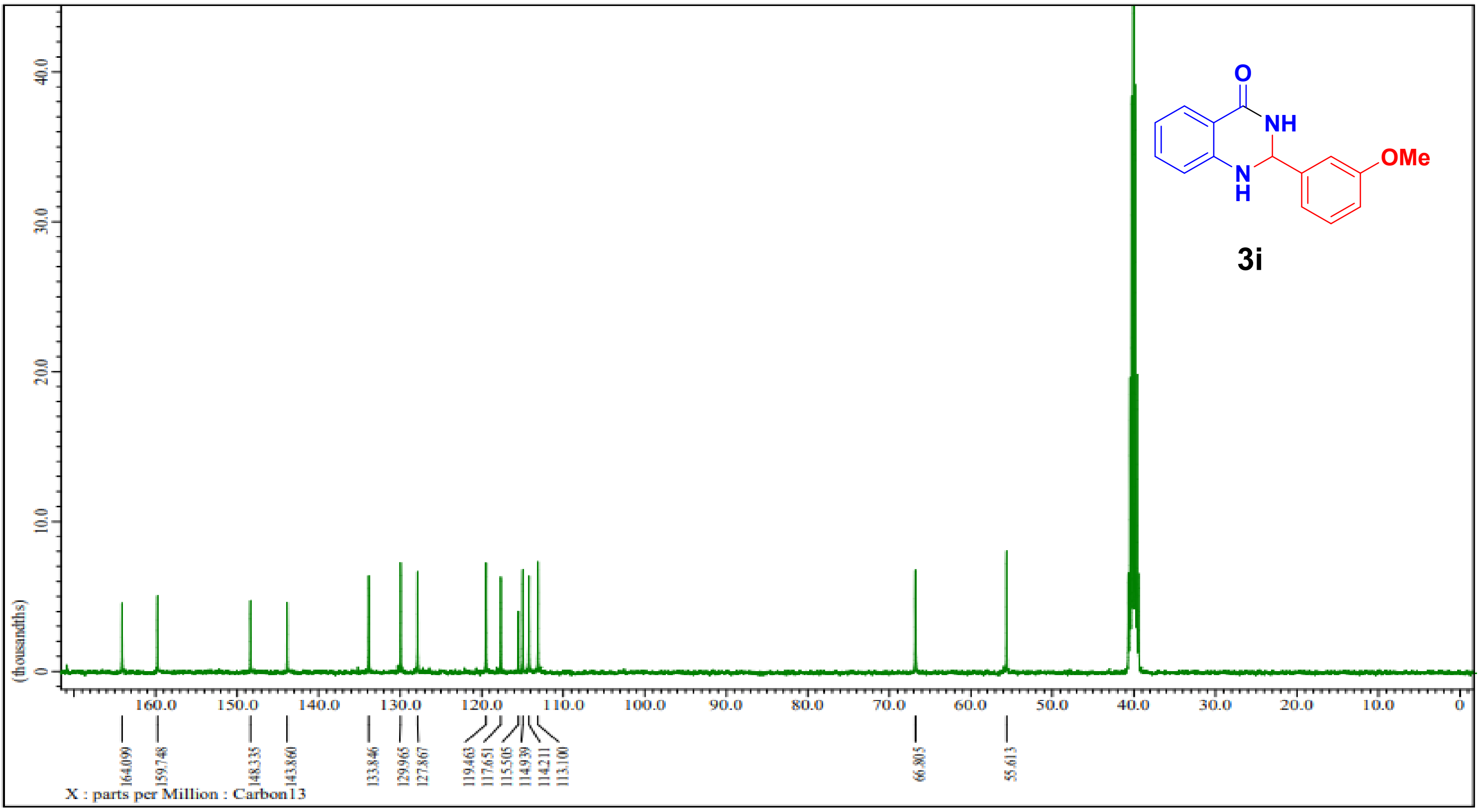
O
NH
N
H
OMe
3i
(thousandths)
164.099
159.748
148.335
143.860
133.846
129.965
127.867
119.463
117.651
115.505
114.939
114.211
113.100
66.805
55.613
X : parts per Million : Carbon13

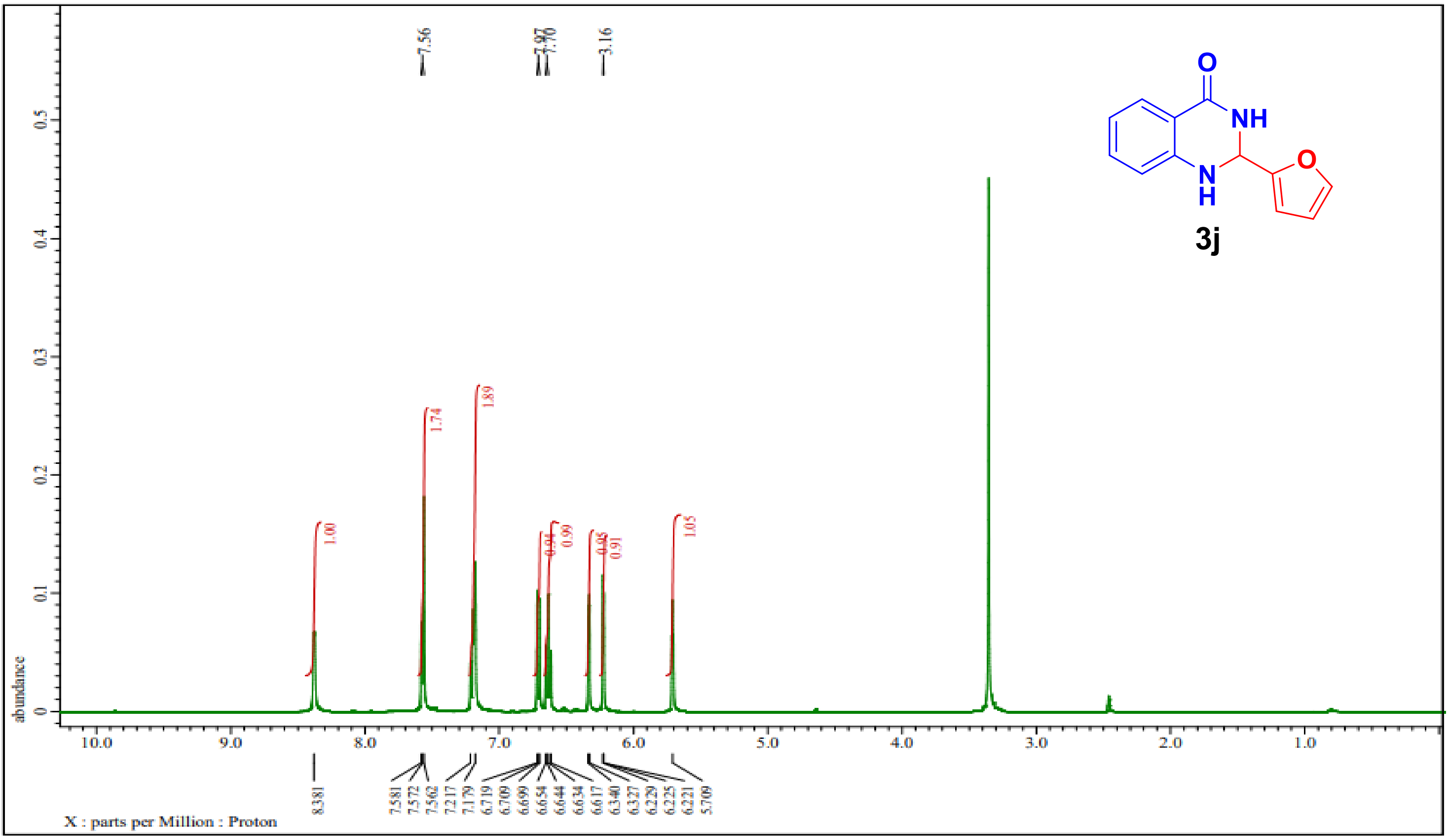

X : parts per Million : Proton
abundance
3j

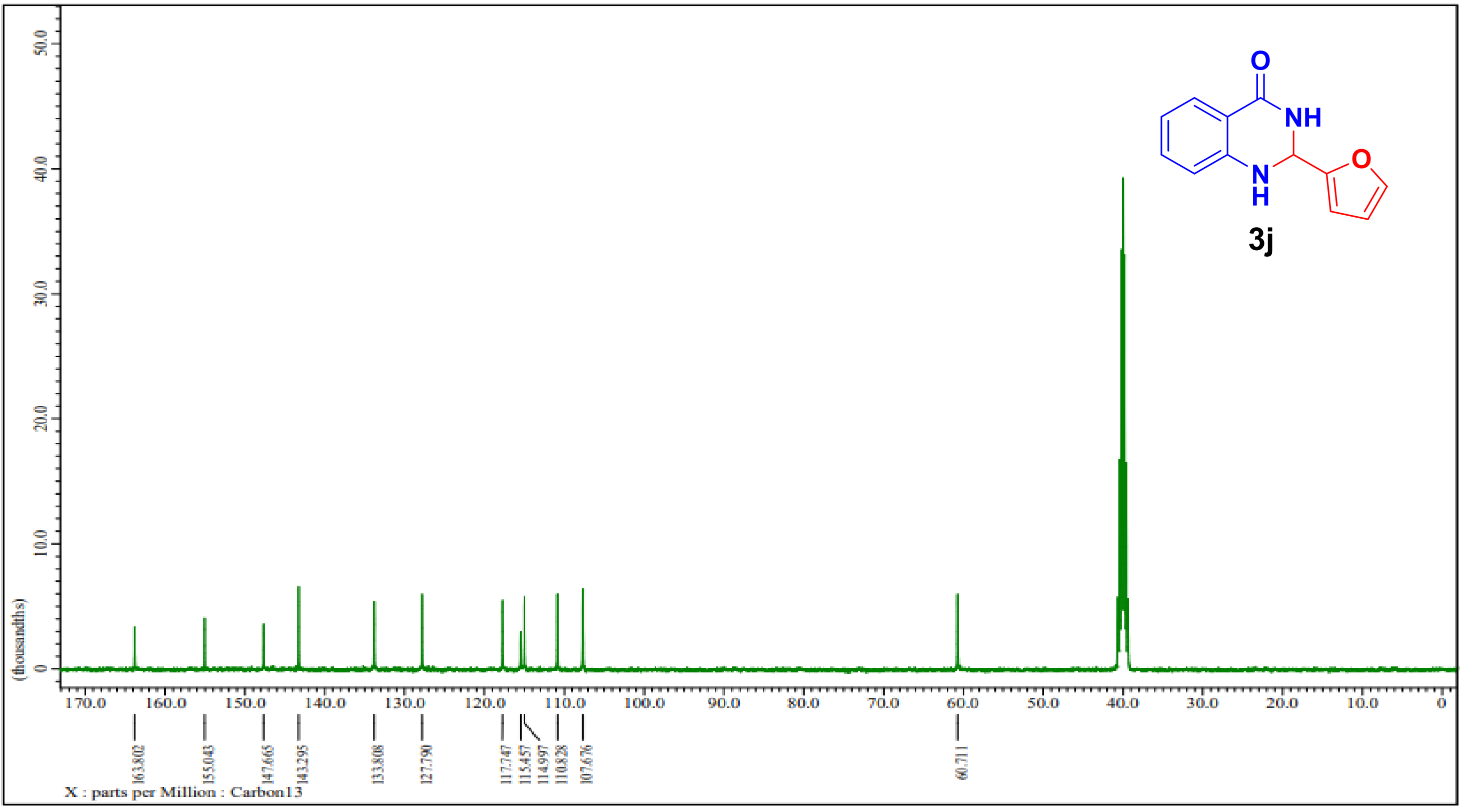

X : parts per Million : Carbon13
(thousandths)
3j

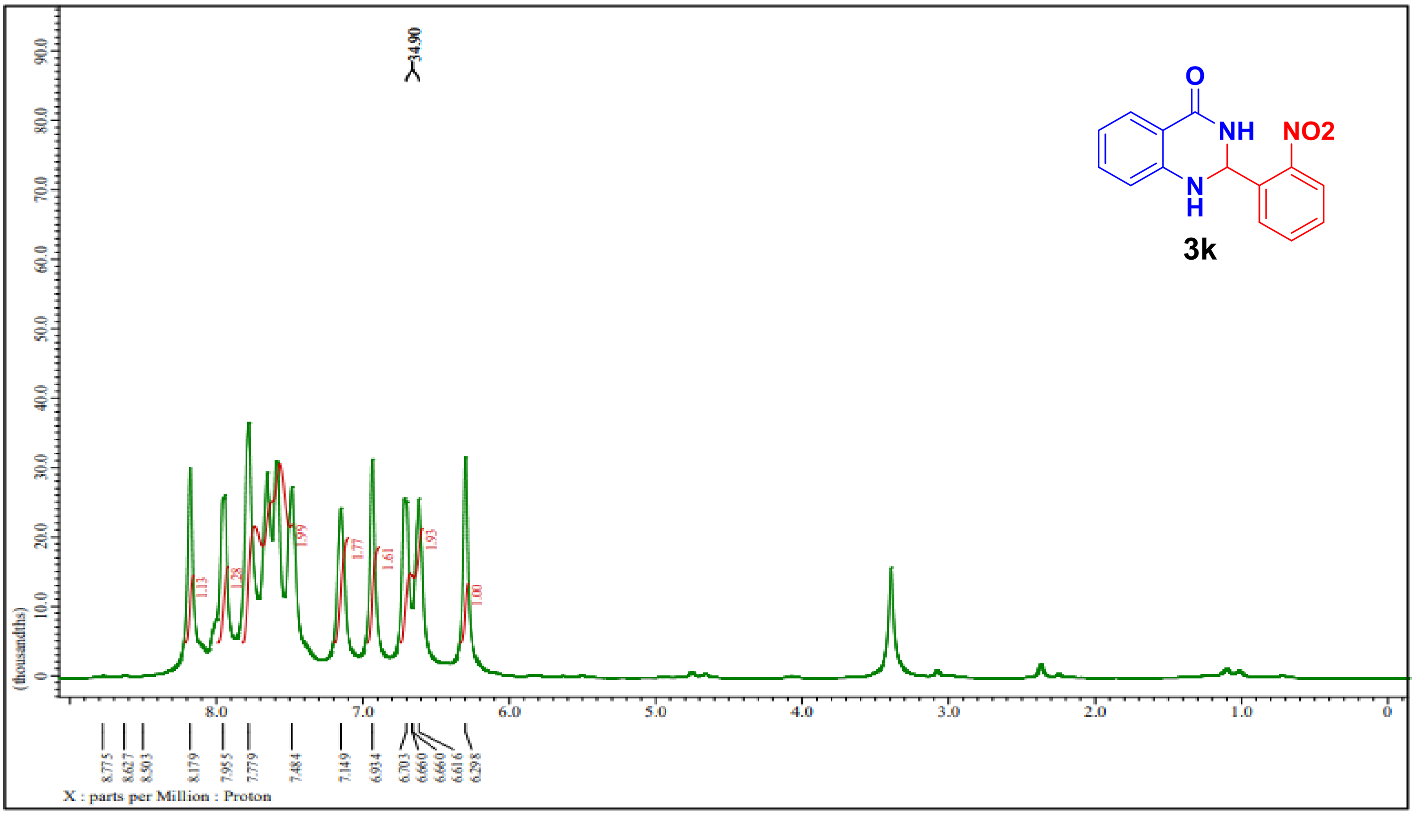

3k
X : parts per Million : Proton
(thousandths)

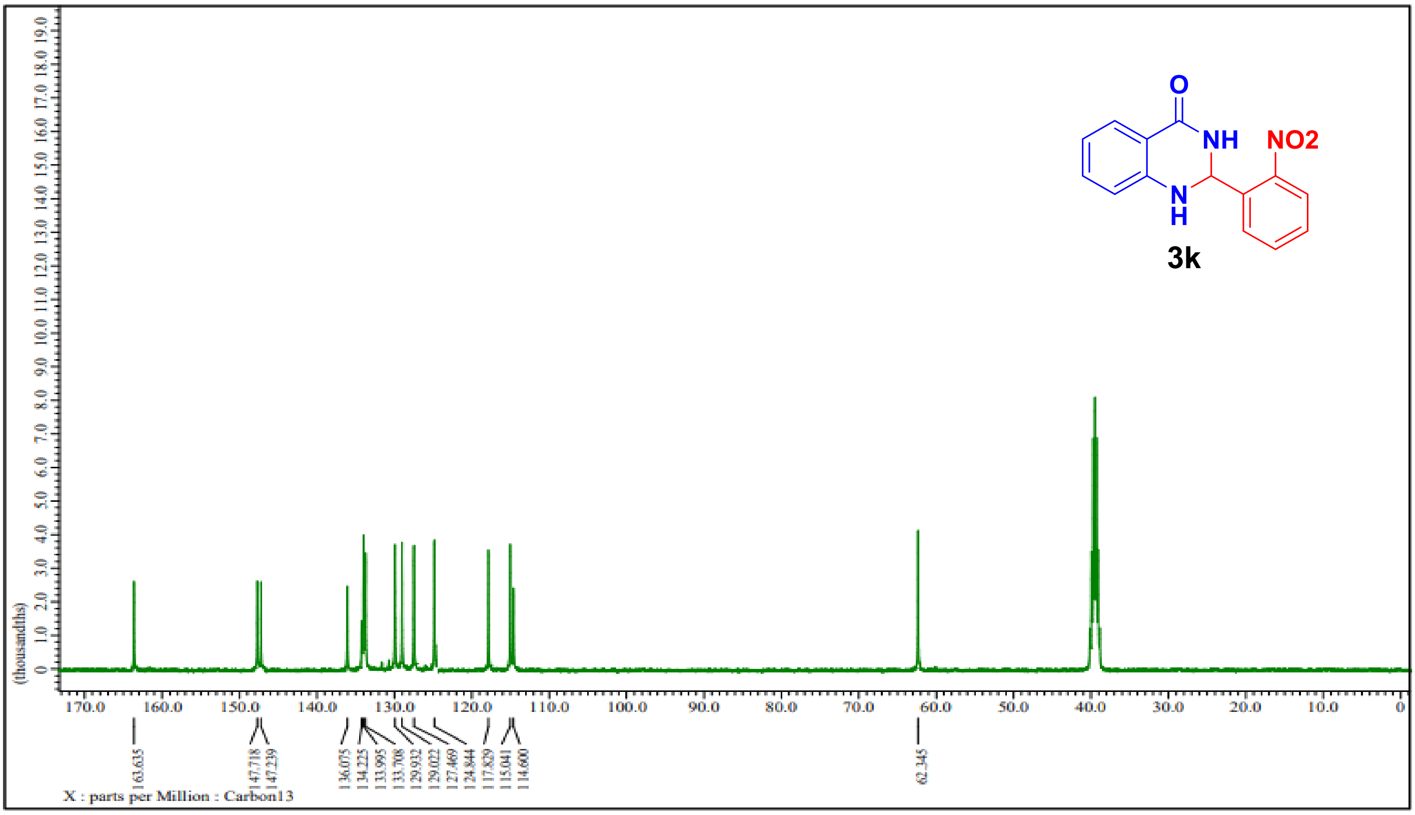

3k
X : parts per Million : Carbon13
(thousandths)

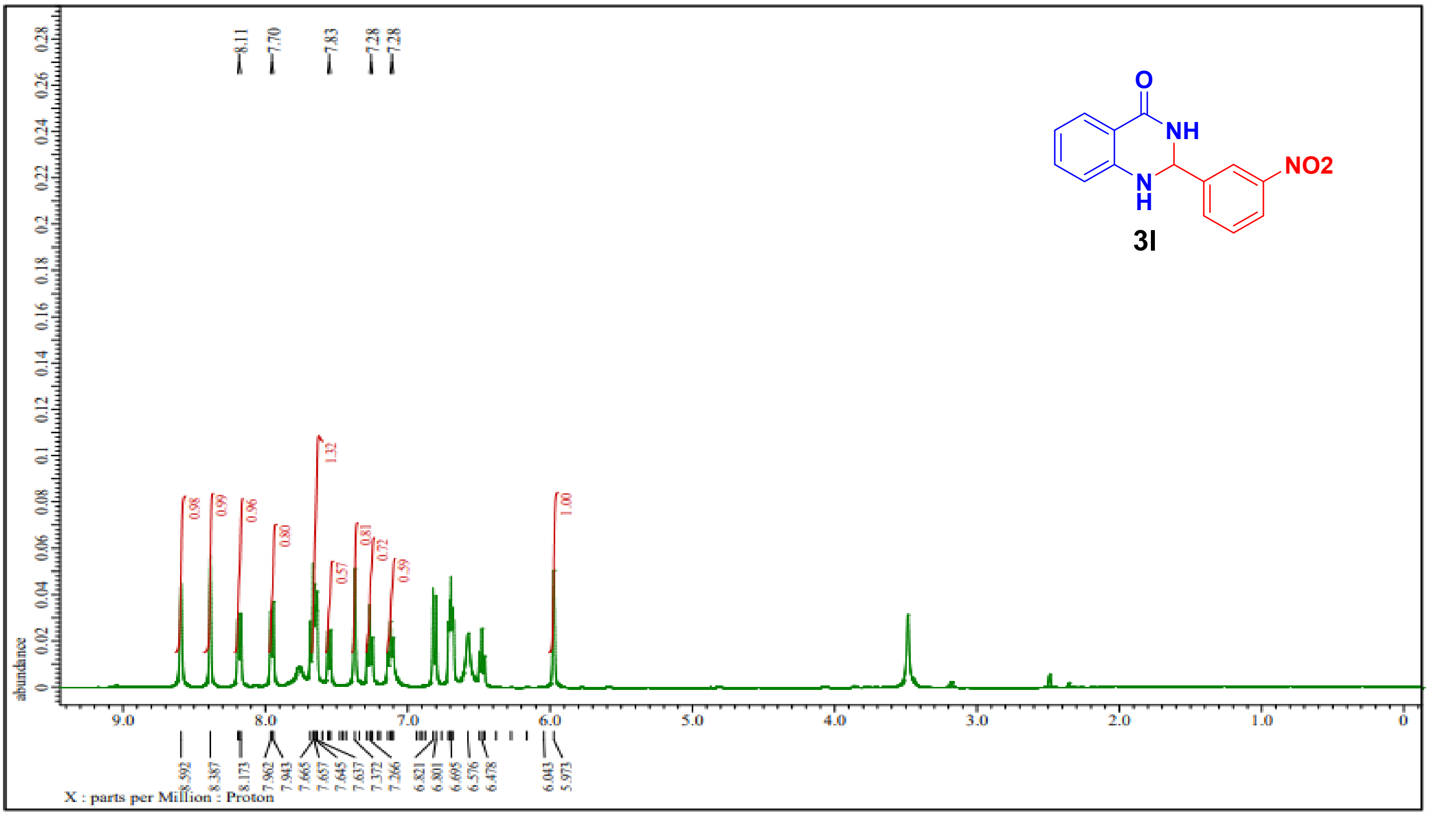
abundance
3l
NO2
NH
N
H
O
8.11
7.70
7.83
7.28
7.28
0.98
0.99
0.96
0.80
1.32
0.57
0.81
0.72
0.59
1.00
8.592
8.387
8.173
7.962
7.943
7.665
7.657
7.645
7.637
7.372
7.266
6.821
6.801
6.695
6.576
6.478
6.043
5.973
X : parts per Million : Proton

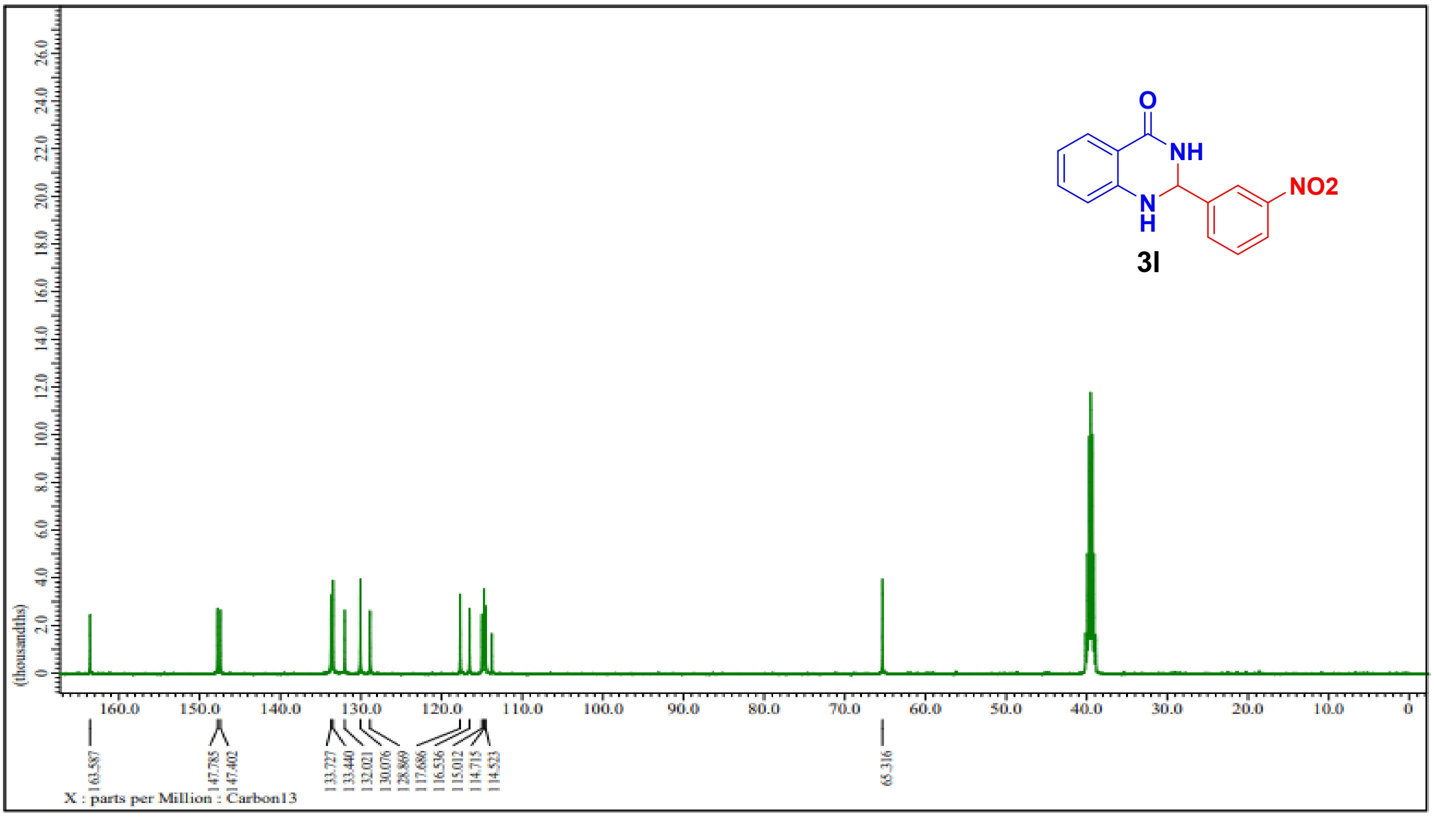
(thousandths)
3l
NO2
NH
N
H
O
163.587
147.785
147.402
133.727
133.440
132.021
130.076
128.869
117.686
116.536
115.012
114.715
114.523
65.316
X : parts per Million : Carbon13
